# Social determinants of health and postnatal wellbeing among women with type 2 diabetes in Thailand: An explanatory sequential mixed-methods study protocol

**DOI:** 10.1371/journal.pone.0354735

**Published:** 2026-07-29

**Authors:** Ratchanok Phonyiam, Tippawan Srichalerm, Arissara Sawatpanich, Jantra Keawpugdee

**Affiliations:** Ramathibodi School of Nursing, Faculty of Medicine Ramathibodi Hospital, Mahidol University, Bangkok, Thailand; Icahn School of Medicine at Mount Sinai, UNITED STATES OF AMERICA

## Abstract

**Introduction:**

Type 2 diabetes mellitus affects pregnancy and can lead to maternal complications. Women affected by diabetes during pregnancy have ongoing physical, psychological, and social needs until birth preparation and after childbirth. The Social Determinants of Health (SDoH) can have a greater impact on health outcomes than healthcare services or personal lifestyle choices alone. To date, no study has explored how women with type 2 diabetes experience life after childbirth. To help close this knowledge gap, the proposed study aims to: 1) Culturally adapt and translate the Postnatal Well-being in Transition scale into Thai. 2) Quantitatively describe the SDoH and postnatal well-being during the diabetes transition among Thai women with T2DM. 3) Explore Thai women’s experiences regarding their SDoH, based on the mean scores of their postnatal well-being during the diabetes transition. And 4) Explore and analyze the impact of postnatal care on women with T2DM using a mixed-methods approach.

**Methods:**

This is an explanatory sequential mixed-methods study protocol with two distinct phases. The first phase involves the translation of the study instrument, while the second phase focuses on data collection for the mixed-methods study. Data will be collected from 50 postnatal women with type 2 diabetes mellitus who have delivered a baby within the past six weeks. Participants will provide data on demographics, social determinants of health, and their postnatal well-being in transition. From this group, 12 women will be selected to participate in semi-structured, one-on-one interviews to gain a deeper understanding of their experiences. The study will use triangulation to integrate and validate the findings from both the quantitative and qualitative data sources.

**Ethics and dissemination:**

Human Research Ethics Committee approval has been received from the Faculty of Medicine Ramathibodi Hospital, Mahidol University. The study findings will be reported in international journals.

## Introduction

As healthcare focuses more on population health and value-based care, addressing social determinants of health (SDoH) has become a key strategy for achieving health equity [1]. Healthy People 2030 defines SDoH as the conditions in the environments where individuals are born, live, work, play, worship, and age [2]. The five key domains of SDoH include economic stability, access to and quality of education, access to and quality of healthcare, the neighborhood and built environment, and social and community context. These factors play a significant role in shaping an individual’s health and well-being [2,3]. Research suggests that SDoH can have a greater impact on health outcomes than healthcare services or personal lifestyle choices alone [4,5]. The relationship between SDoH and diabetes care during pregnancy is significant, particularly disparities in maternity care [6].

Type 2 diabetes mellitus (T2DM), among other chronic conditions, affects pregnancy and can lead to maternal and neonatal complications [7]. While extensive global research exists on how SDoH impact diabetes in adults, there is a specific need to understand these factors in Asian populations. In Thailand, for instance, a prior study revealed that pregnant women with T2DM face unique challenges in managing their condition. These include difficulties with food cravings, episodes of hypoglycemia, avoiding physical activity due to fear of miscarriage, and limited health literacy concerning insulin injections [8]. These challenges may not only occur during pregnancy but may also persist during childbirth and the postnatal period [9,10]. Women with different SDoH backgrounds may face unique challenges and require needs.

The transition to motherhood is especially critical for women with T2DM. After delivering the baby, they often experience breastfeeding difficulties, such as delayed lactation—particularly when the baby is separated from the mother due to neonatal health issues like neonatal hypoglycemia and the Neonatal Intensive Care Unit (NICU) admission [11]. Additionally, in the early postnatal period, those women may struggle to breastfeed while maintaining reasonably stable blood glucose levels [12,13].Health professionals play a vital role in supporting women with diabetes, yet many women report that their healthcare providers do not offer adequate knowledge, skills, or support during the transition to motherhood. During this transition, women with diabetes seek advice and support from health professionals who can communicate meaningfully, recognize their self-management efforts, treat them as equals, and show empathy, understanding, and respect [8]. To date, no study has explored how women with type 2 diabetes experience life after childbirth. This study aims to describe the social determinants of health and postnatal well-being during the transition for postnatal women with T2DM. The research will help fill the gap in knowledge and make postnatal care more effective and tailored to the needs of women with type 2 diabetes. Thus, identifying women’s SDoH and gaining a better understanding of their experiences with postnatal care will make it more effective and better suited to the needs of women with type 2 diabetes.

Such exploration is necessary because we need to not only provide care for pregnant women with diabetes during their pregnancy, but also understand their preparation for birth and beyond, into the postnatal period. Women affected by diabetes in pregnancy have ongoing physical, psychological, and social needs [14]. A study from a multi-ethnic, low socioeconomic women population showed that preparation for birth and postnatal diabetes follow-up were areas requiring significant improvement [15]. These challenges were mitigated through care from a consistent diabetes care which extended support to both the mother and her baby [15].

To address this knowledge gap, the present study employs an explanatory sequential mixed-methods design [16]. This design enhances the understanding of postnatal well-being by systematically integrating quantitatively derived patterns with women’s lived experiences. The quantitative component provides generalizable evidence on the associations between social determinants of health and postnatal well-being during the postpartum diabetes transition among Thai women with type 2 diabetes, while the qualitative component offers in-depth contextual insights into how and why these associations manifest in everyday life.

The integration of both phases enables the identification of convergences, divergences, and complementary explanations that may not be fully captured through a single-method approach. Building on this framework, the study is structured around four objectives: (1) to culturally adapt and translate the Postnatal Well-being in Transition instrument into Thai; (2) to quantitatively examine the social determinants of health and postnatal well-being during the postpartum diabetes transition; (3) to qualitatively explore women’s lived experiences of social determinants of health in relation to their postnatal well-being scores; and (4) to integrate findings from both phases to generate a comprehensive assessment of postnatal well-being in this population.

Overall, this mixed-methods approach facilitates a more nuanced and comprehensive understanding of postnatal well-being among Thai women with type 2 diabetes, thereby informing the development of culturally appropriate, evidence-based interventions and health policies aimed at improving postpartum outcomes.

## Methods

### Study design

This is an explanatory sequential mixed-methods study protocol to be conducted in Thailand. The purpose of an explanatory sequential design is to expand and elaborate on the study findings from the quantitative strand through qualitative methods in the second strand to better understand the social determinants of health and postnatal wellbeing among women with type 2 diabetes (Table 1).

**Table 1:** Explanatory sequential mixed methods flow chart diagram.

Phase	Process	Details
**Quantitative Phase**	Questionnaires translation	• Translate materials into Thai • Back translation into English to see two versions compatibility • The research team review the Thai versions for clarity and cultural appropriateness and revised as need.
	Questionnaires’ content validity index by three experts	• Three experts who are actively in women’s health will validate the questionnaires before implementation
	Quantitative study	• Cross-sectional design • Self-reported questionnaires via REDCap software
	Quantitative data analysis	• Data screening • Descriptive statistics (percentage, frequency, means, standard deviation) using SPSS software
**Qualitative Phase**	Interaction point #1 and data collection	• Use the quantitative results to inform adjustment of initial interview questions and probes. • Interview guide: 12 participants will be joining semi-structured interviews
	Qualitative study	• Individual, phone, semi-structured interviews following interview guide • Audio-recorded and transcribed
	Qualitative data analysis	• Coding using inductive/deductive hybrid thematic analysis • Atlas.ti software
**Integrated results**	Integration point #2: interpretation of quantitative and qualitative results	• Integration and explanation of quantitative and qualitative results • Joint display of quantitative and qualitative data • Develop meta-inferences from data integration • Conclusion the results

### Research setting

This study will be conducted across two public hospitals in Thailand—one urban and one suburban. The urban site is a tertiary care center in Bangkok, serving a high-density population with diverse socioeconomic backgrounds, ranging from low to high income. In contrast, the suburban hospital in Samut Prakan serves a more stable, residential, and primarily middle-income community. This dual-site approach utilizes maximum variation sampling to capture a diverse range of experiences, drawing on participants from both urban and rural settings to reflect their distinct contexts. By selecting sites with contrasting geographical and socioeconomic characteristics, the study aims to account for the varying SDoH that influence maternal well-being during the postpartum transition.

This study is divided into two phases: 1) translation of the instrument and 2) data collection for the mixed-method study.

## Part 1: Translation tool

Written authorization to translate, adapt, and validate the *Postnatal Well-being in Transition* questionnaire was granted by the developer of the original tool. We followed the six-stage translation process approach developed by Beaton and colleagues in 2000 [17]. For its use in the Thai population, we culturally translated and adapted the questionnaire into Thai, then back-translate it to ensure clarity and cultural appropriateness. For the forward translation phase, two bilingual Thai-English nursing faculty members independently translated the instrument from English to Thai, with careful consideration given to cultural appropriateness (Forward 1 and Forward 2). The research team then reviewed both versions to produce a synthesized third version (Forward 3). Subsequently, the Forward 3 was sent to two different bilingual Thai-English nursing faculty members for independent back-translation from Thai to English (Back 1 and Back 2). Finally, the research team convened a meeting to resolve any discrepancies and establish the final Thai version of the ‘Postnatal Well-being in Transition’ scale prior to validation.

The questionnaire was validated by three experts in postnatal care in Thailand, and the content validity index was calculated before implementation with Thai women with type 2 diabetes. The Item-level Content Validity Index (I-CVI) was calculated as the proportion of experts who gave a rating of 3 (quite relevant) or 4 (very relevant). The Scale-level Content Validity Index (S-CVI), specifically the average scale-level index (S-CVI/Ave), was calculated by summing the I-CVIs of all items and dividing by the total number of items. Any item receiving a score of 1 (not relevant) was considered unacceptable and removed from the scale. Items requiring modification based on expert feedback were revised, and all such adjustments are reflected in the final version of the instrument. Validation has been completed; the I-CVI and S-CVI were both 1.0, as determined by a panel of three experts.

## Part 2: Study data collection

### Eligibility criteria

#### Inclusion criteria.

Postnatal women with type 2 diabetes mellitus who have delivered a baby within the past 6 weeks [18], either primigravida or multigravida; aged 20–44 years; are able to speak Thai; and are able to provide consent. Participants will be eligible for inclusion regardless of infant clinical status (including, but not limited to, NICU admission, congenital anomalies, stillbirth, or neonatal death).

#### Exclusion criteria.

Pregnant women with type 1 diabetes mellitus (T1DM) and gestational diabetes mellitus (GDM) as well as those with health complications, such as heart disease, chronic kidney disease, or other conditions affecting hearing or vision that may be indicative of long-term diabetes and require advanced diabetes management, will be excluded from the study.

Potential participants will be identified at urban and suburban hospital sites through medication records and clinical visits during the postnatal period. Interested individuals will be directed to a secure online platform (Research Electronic Data Capture: REDCap), where they will view the online informed consent form. Participants must pass a brief ‘consent check’ question before the system allows them to proceed to the survey, ensuring they have read and understood the study requirements. We will monitor non-response rates by tracking the number of individuals who do not wish to complete the survey. Additionally, mode equivalence will be monitored to ensure that data collection remains consistent across both urban and suburban settings.

Translation of the tool has been completed. Participant recruitment and data collection commenced in September 2025. We expect to complete data collection by December 2026, and the results are projected to be finalized by June 2027.

### Quantitative phase

As this study is descriptive rather than comparative in nature, statistical significance does not drive the determination of target sample size. This study will recruit 50 participants (Maghalian et al., 2024) through both online methods and face-to-face outreach at postnatal units in two public hospitals.

#### Recruitment.

Participants for the survey will be recruited from postnatal units in two public hospitals in Thailand. The recruitment process will involve contacting the head nurse of each postnatal unit to describe the study objectives and request permission for data collection. Once permission is granted, the head nurses will distribute the advertisement document in the form of an e-poster to postnatal women who are hospitalized in their unit.

### Qualitative phase

A total of 12 women will be contacted for qualitative interviews: six participants (n=6) with the lowest mean total scores (indicating lower well-being) and six participants (n=6) with the highest mean total scores (indicating higher well-being). Semi-structured phone interviews will be conducted to gain a deeper understanding of postnatal well-being during the transition to motherhood. The sample size of qualitative arm was guided by the previous study among Thai pregnant women as data saturated at 12 participants [8]. If data saturation is not reached by the 12th interview, we will continue interviewing participants from both the lower and higher well-being groups accordingly until no new themes emerge.

The qualitative component utilized three types of purposeful sampling: nested. Nested sampling, a feature of explanatory sequential mixed methods designs, involves re-sampling the same individuals who participated in the quantitative phase for the qualitative phase. As a result, the same inclusion and exclusion criteria from the quantitative phase were applied to the qualitative phase [16]. The use of a nested approach is intentional, as this inter-relationship is a recognized strength of the explanatory sequential design; it allows for a deeper understanding of the extreme cases identified in the first phase.

This study’s qualitative research used purposeful sampling, supported by nested sampling, to select information-rich cases. Purposeful sampling involves intentionally choosing specific units or cases based on a clear objective, rather than random selection, and it can be used to achieve representativeness or comparability [16]. This method allowed for a broader range of cases to be selected and facilitated comparisons across different types. Results from the quantitative phase were examined to inform the qualitative sampling strategy, with the potential to apply stratified and theoretical sampling techniques. Stratified sampling helped identify subgroups and enabled comparisons between them, which aligned with the qualitative research question. It was particularly valuable as it captured variations in the phenomenon of interest. To apply this, participants from the quantitative phase were stratified based on postnatal well-being scores during their transition. This approach aims to explore the challenges these women experience that contribute to their reduced well-being during the postnatal period as they adjust to motherhood.

#### Recruitment.

Participants for the semi-structured interviews will be recruited from the same pool of postnatal women who participated in the survey. They will be invited to participate in the interviews via online platforms (1) Facebook Page: Chat about Mom and 2) Phone call) or direct communication (meet in-person at data collection sites), providing them with the opportunity to further contribute to the study through in-depth discussions.

### Quantitative data collection

Three questionnaires are as follows:

First, we will collect demographic data using a questionnaire, which will include the mother’s age, marital status, duration of diabetes, parity, date of delivery, gestational age at delivery, and current medications.

Second, we will assess the social determinants of health across five domains [3]. The questionnaire for this measure is based on previous research and evidence [6]. This 20-item tool consists of two open-ended questions and 18 frequency-based items designed to quantify women’s experiences with social determinants of health (SDoH). There is no formal scoring system for this tool; the responses will be rated as frequency of each item. Validation has been completed; the I-CVI and S-CVI were both 1.0, as determined by a panel of three experts.

Domain 1: Economic Stability (4 items)– Family income and employment status.
Domain 2: Education Access and Quality (1 item)– Educational attainment.
Domain 3: Health and Healthcare (4 items)– Health insurance, tobacco use, alcohol use, and comorbidities.
Domain 4: Neighborhood and Built Environment (7 items)– Transportation and access to healthy foods
Domain 5: Social and Community Context (4 items)– Unsafe neighborhoods and community interaction

Third, we will measure postnatal well-being using the *Postnatal Well-being in Transition* questionnaire [18]. This 30-item questionnaire, which uses for each item a 5-point Likert scale from 1 (strongly disagree) to 5 (strongly agree).

### Data analysis

For quantitative data, information from questionnaires will be entered and analyzed using IBM SPSS version 25.0. The analyses include calculating means, standard deviations, and ranges for continuous variables and frequencies and percentages for categorical variables. Missing values will be replaced using item mean imputation; any missing value for a participant will be substituted with the mean value calculated from all available responses for that variable [19].

### Qualitative data collection

We have developed the interview guide which aims to deepen the understanding of women’s experiences during their transition to motherhood. The interviewer is a female nursing faculty member with 12 years of clinical nursing practice and expertise in qualitative data collection. At the start of each interview, she explicitly states that she is acting in a research capacity, rather than a clinical one, to encourage honest disclosure. Interviews are conducted over the phone at times most convenient for the participants. A semi-structured interview guide is used to allow participants to lead the narrative, ensuring their lived experiences take precedence over the researcher’s clinical assumptions. The interviewer records field notes regarding the conversational tone, date, and time of each interview.

### Data analysis

Since the sampling is sequential, using quantitative scores to identify participants, the analysis of the qualitative interviews will be conducted independently. Coders will be blinded to the specific individual quantitative scores of the participants during the coding phase.

Interview transcripts will be analyzed using Atlas.ti version 9 for qualitative data analysis, which will facilitate data organization and joint analysis of the text. We will identify recurring patterns in participants’ responses based on their relevance to the predetermined domains of inquiry. Descriptive coding will be employed to assign a single code to each segment of data (2–3 lines) that conveys a complete, standalone meaning. The analysis will involve a continuous process of moving back and forth between the entire dataset and individual transcript coding. The coders will work systematically through the dataset, giving equal attention to each transcript and identifying interesting aspects that could form the basis of recurring patterns across the dataset. They will then describe a clear definition and name for each theme by identifying its essence and determining the aspects that capture information. A table will be developed to present the themes, subthemes, and quotations.

### Data management

Quantitative data will be stored in the REDCap database. Participants will be tracked using ID numbers. The file linking participant IDs to their contact information will be stored in a separate secure location. A research assistant will transcribe the audiotape verbatim in the Thai language. All information, such as pauses and laughter, will be noted in each transcribed interview. All identifiable information, such as participants’ names and hospital settings, will be removed. Each interview file and questionnaire will be labeled with a three-digit number in order (e.g., ID001). Participants will be tracked using case identification numbers. Interview data (audio files) and transcribed interviews will be saved on a secure server. Assurances of protection of individual participant privacy and confidentiality will be provided. Data results will be reported only in aggregate form.

New data will be obtained through surveys and interviews conducted with Thai postnatal women with T2DM. This data will include demographic details, survey responses, interview transcripts, and any other relevant information related to the study’s objectives. To ensure quality control and reproducibility, the data will be systematically organized, meticulously documented, and clearly described. Standardized formats and protocols will be followed throughout. Data will be securely stored in password-protected folders on a Mac computer, with regular backups conducted to reduce the risk of data loss. The principal investigator will be responsible for managing the data throughout the research process. All data will be retained for 3 years, after which it will be deleted from password-protected folders on a Mac computer and its backup files.

The findings of this study will be published in an international peer-reviewed journal. To protect participant confidentiality and privacy, the de-identified datasets generated during the study are not publicly archived but are available from the corresponding author upon reasonable request.

### Mixed-methods approach: Triangulation of qualitative and quantitative data

Triangulation is desirable in mixed-methods research because it serves to validate and confirm the phenomena being studied [20]. To conduct triangulation, qualitative and quantitative analyses will be conducted separately. The results will then be compared and contrasted to identify data convergence and divergence. To ensure unbiased triangulation, a side-by-side comparison will be conducted during the final integration phase. We will identify exemplary quotations where qualitative themes either converge with or contradict quantitative trends.

Statistics-by-themes joint displays organize quantitative results (e.g., high and low well-being) across qualitative themes in a matrix format, resembling a crosstabulation. These displays feature qualitative themes in the first column, paired with corresponding statistical data (means and standard deviations). This layout enables the research team to triangulate data, identifying areas of convergence or divergence between the two sources.

To transition from a descriptive joint display to an analytical integration, the research team will follow the three-step interpretive framework [21] to derive integrated meta-inferences.

Step 1: Systematic Assessment of Fit. Once quantitative metrics (means, percentages) and qualitative data (themes, quotes) are populated side-by-side in the joint display, the team will evaluate their relationship using three analytical criteria: *Confirmation (Convergence):* Qualitative findings directly validate or mirror statistical trends. *Expansion:* Qualitative data explains or nuances a statistical trend (e.g., high quantitative well-being scores contrasted by qualitative insights showing mothers suppress pain to prioritize childcare). *Discordance (Divergence):* Quantitative metrics and qualitative findings present conflicting realities.
Step 2: Procedural Resolution of Discordance. In instances of discordance, the team will initiate an analytical reconciliation process (Fetters et al., 2013) by: (1) checking raw quantitative data for outliers or measurement biases; (2) re-examining the qualitative matrix for sub-demographic patterns; and (3) conducting peer debriefings to explore whether the instruments measured different dimensions of the same phenomenon.
Step 3: Synthesis into Meta-Inferences. Finally, the team will synthesize the reconciled findings into overarching meta-inferences. Documented in the final column of the joint display, these meta-inferences ensure the discussion yields a fully integrated, multi-dimensional understanding of postnatal well-being rather than parallel independent findings.

### Ethics and dissemination

This protocol has been reviewed and approved by the Faculty of Medicine Ramathibodi Hospital, Mahidol University, Thailand (MURA.2025/648).

### Quantitative phase

#### Informed consent.

Participants will be provided with a detailed study description along with the survey link or QR code. They will be informed that participation is voluntary and that by completing the survey, they are providing consent to participate in the study. The survey introduction will emphasize the confidentiality of their responses and assure them that their names will not be used or reported in any way. Participants will have the option to decline participation at any time without consequence.

#### Time for consideration.

Participants will be given sufficient time to consider their decision to participate before proceeding with the survey. There will be no time pressure, and participants can choose to complete the survey at their convenience. The survey will take approximately 30–45 minutes.

### Qualitative phase

#### Informed consent.

Prior to the phone interview, participants will receive a detailed study description outlining the purpose and procedures of the interview. They will also be provided with an informed consent form, which they will be asked to review and sign before the audio-recorded interview begins. The informed consent form will reiterate the voluntary nature of participation, confidentiality measures, and the right to withdraw from the study at any time.

#### Time for consideration.

Participants will be given adequate time to review the study information and informed consent form before the interview. They will have the opportunity to ask any questions they may have about the study or the interview process before providing their consent. The interview will take approximately 30–45 minutes.

The Thai version of the *Postnatal Well-being in Transition* scale will be made available upon request. The development of the project and its findings will be reported in relevant international journals.

### Limitation

A key limitation of this study is the lack of formal cross-cultural psychometric validation of the adapted Social Determinants of Health (SDoH) instrument within the Thai population. While the tool demonstrated strong content relevance via a Content Validity Index (CVI) from an expert review panel, content validity alone does not guarantee cultural equivalence. Because this instrument was adapted from Western framework domains, it may not fully capture distinct Thai sociocultural nuances. Future research should prioritize the formal translation, cultural adaptation, and psychometric validation of standardized SDoH metrics tailored specifically to Southeast Asian populations.

### Research significance, impact, and outcomes

The protocol outlines a study designed to improve our understanding of the social determinants of health and well-being for Thai women in the postnatal period. A mixed-methods approach is crucial for this, as it allows us to fully capture the complexity of women’s experiences by combining qualitative and quantitative results.

The findings have significant implications for the future of nursing research. They highlight the need for future studies on pregnant women with T2DM to use mixed methods designs, which can illuminate the evolution of the postnatal journey. By gaining a better understanding of a woman’s unique healthcare needs, we can create better tools for assessing women’s postnatal health based on their specific social determinants. This knowledge of sociocultural factors will eventually help us design patient-centered, culturally tailored care to improve women’s health outcomes. Furthermore, our findings could be used as a model for developing new healthcare guidelines in Asian regions and will eventually inform policy decisions, leading to a more integrative care model that benefits both mothers and infants in Thailand.

## Supporting information

S1 Fig An overview of the explanatory sequential mixed method design. (TIFF)

S2 Fig Study timeline. (TIFF)
