# Supplementary figures and images for: Social determinants of health and postnatal wellbeing among women with type 2 diabetes in Thailand: An explanatory sequential mixed-methods study protocol
Source: PLoS One. 2026 Jul 29;21(7):e0354735. doi: 10.1371/journal.pone.0354735 (PMC13419172; doi:10.1371/journal.pone.0354735)

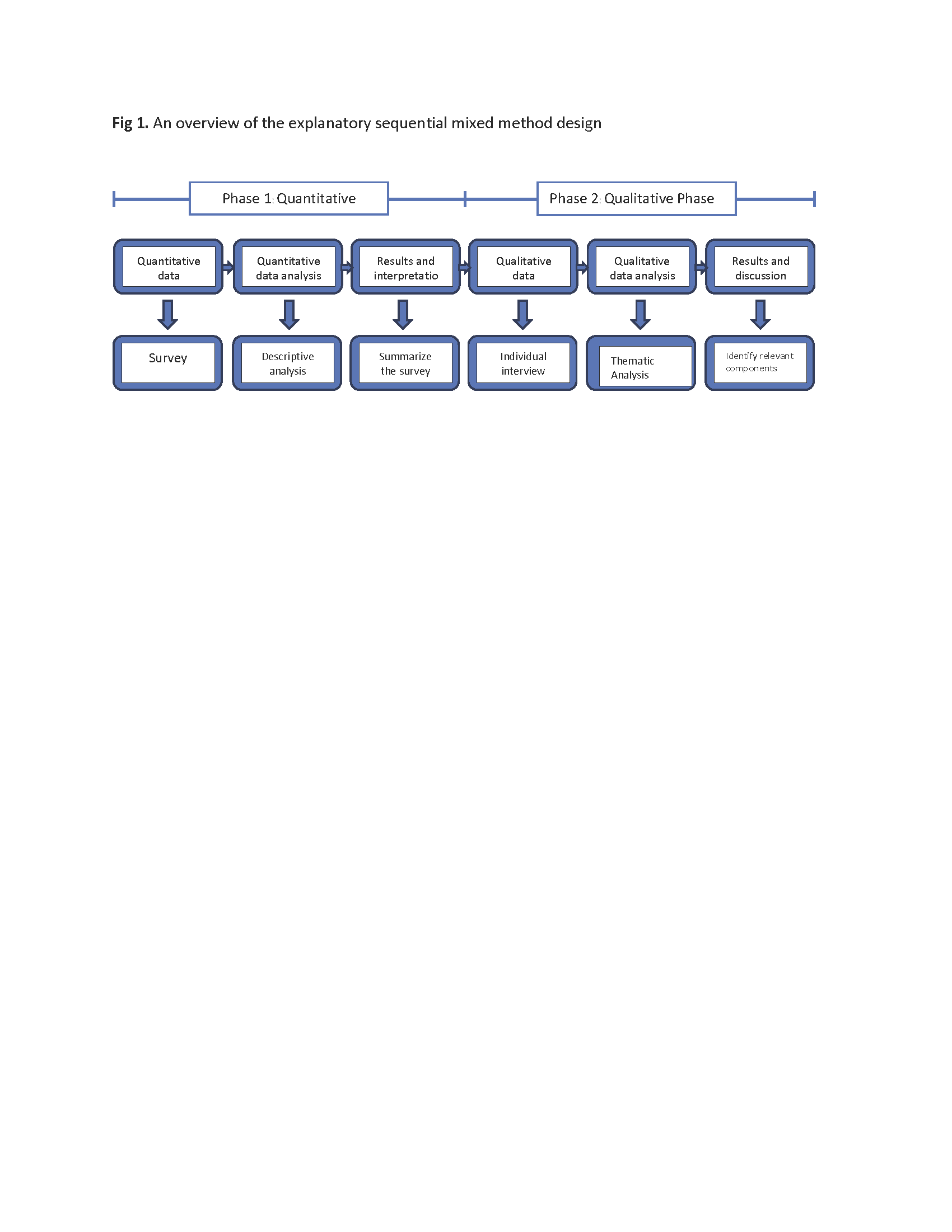

Supplement: S1 Fig — (TIFF) [file pone.0354735.s001.tiff]

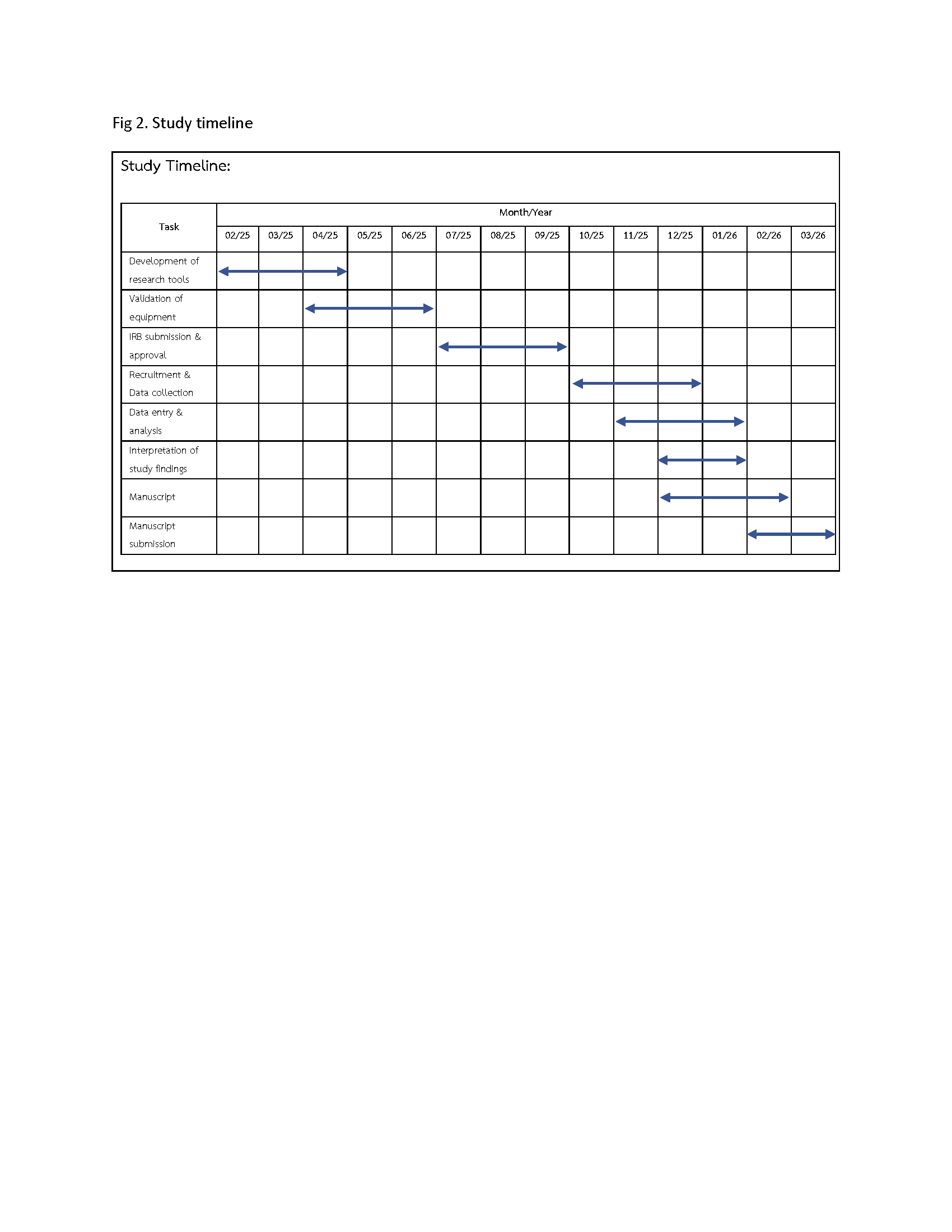

Supplement: S2 Fig — (TIFF) [file pone.0354735.s002.tiff]
