# Supplementary material for: Social determinants of health and postnatal wellbeing among women with type 2 diabetes in Thailand: An explanatory sequential mixed-methods study protocol
Source: PLoS One. 2026 Jul 29;21(7):e0354735. doi: 10.1371/journal.pone.0354735 (PMC13419172; doi:10.1371/journal.pone.0354735)
Supplement: Attachment — Submitted filename: Review Manuscript PONE-D-25-52097.pdf [file pone.0354735.s003.pdf]

REVIEW COMMENTS

MANUSCRIPT NUMBER: PONE-D-25-52097

This manuscript addresses a highly relevant and meaningful topic, focusing on social determinants of health and postnatal well-being among women with type 2 diabetes, an area where further evidence is clearly needed to inform clinical practice and health policy. The study has the potential to contribute valuable insights, particularly given its mixed-methods approach and its focus on a specific population and context. My comments are provided in the spirit of strengthening the manuscript, with particular emphasis on improving clarity of reporting, methodological transparency, and reproducibility. These aspects are especially important in protocol papers, as they allow readers to understand the planned study in detail and to assess any deviations when the final results are published. For clarity, my comments and recommendations are organized into two sections: (1) Introduction and (2) Methods. Each section concludes with a summary of key recommendations to facilitate revision.

A) INTRODUCTION

Specific comments

<u>Comment 1:</u>

The opening statement (lines 58 and 59) requires clarification, particularly given its position as the first sentence of the manuscript.

It conflates three distinct components: (1) a shift toward population health/value-based care, (2) SDOH as a "key strategy," and (3) achieving health equity as an outcome. It does not specify where, according to whom, or based on what type of evidence these claims are made, which makes the statement too broad and insufficiently contextualized.

Regarding the supporting citation for this opening statement, the manuscript relies on reference 1 to support this statement. However, reference 1 is a narrative scientific review reflecting expert consensus, not primary empirical evidence. Moreover, the specific statement cited in reference 1 is not based on direct empirical findings within that paper; instead, it draws on other sources, mainly policy documents (e.g., CMS Equity Plan, HHS Action Plan, Chin 2016), which the authors may wish to consult directly to better align the citation with the intended claim.

As a result, the manuscript is citing a conceptual statement from a review article that itself relies on policy-oriented references, rather than directly citing empirical or primary evidence. This creates a mismatch between the strength of the claim made in the manuscript and the type of evidence used to support it, and may give the impression that the statement is more strongly evidence-based than is actually demonstrated by the cited source.

I recognize that these claims are supported by empirical evidence in the broader literature and are consistent with current understanding. However, because this is a strong and important statement, particularly as the opening sentence of the manuscript, it requires greater accuracy and precision in how it is phrased and supported. It would be more appropriate to cite direct empirical or policy-level references that substantiate each component of the claim, rather than relying indirectly on a conceptual statement from a narrative review article.

Comment 2

The reference cited for the definition of social determinants of health (lines 60 to 64) appears to be a website and is not formatted according to PLOS citation guidelines (e.g., missing standard elements such as organization, publication details, and access date). The authors should revise this citation to comply with the required format.

Additionally, the authors may consider citing more authoritative or primary sources for this definition, such as official reports, white papers, or position statements. In particular, the concept of social determinants of health has been more broadly and originally established by the World Health Organization (e.g., WHO Commission on Social Determinants of Health), whereas the Healthy People 2030 framework reflects a U.S.-specific adaptation. Using a more foundational or internationally recognized source may strengthen the conceptual grounding of the manuscript.

Comment 3

In the manuscript, the statement “SDoH can have a greater impact on health outcomes than healthcare services or personal lifestyle choices alone” (lines 64 to 66) is supported by reference 3. However, reference 3 is a Commentary-type article that does not present original empirical data but instead cites other studies to support this claim. While the underlying evidence exists, the manuscript is relying on a secondary source that summarizes this evidence, rather than citing the primary empirical studies directly.

In addition, the primary purpose of the cited paper is not to evaluate or demonstrate this claim, but to discuss the need for data science methods and capacity in the study of SDoH.

Therefore, the reference is not fully aligned with the specific statement it is being used to support.

For a statement of this strength, it would be more appropriate to cite the original empirical studies that provide the supporting evidence and are more directly aligned with the claim.

Comment 4

The statement "Additionally, in the early postpartum period, those women may struggle to breastfeed while maintaining reasonably stable blood glucose levels" (lines 81 and 82) is supported by reference 10; however, this paper primarily focuses on the validation of a questionnaire (DBM-Q) rather than providing direct empirical evidence quantifying this specific difficulty. While the introduction acknowledges that women may face challenges managing breastfeeding and glycaemic variability, the study itself is not designed to substantively evaluate or demonstrate this claim. Therefore, the reference only provides indirect contextual support, and a more appropriate citation would be a study specifically investigating breastfeeding-related glycaemic instability in the early postpartum period.

General comments

The introduction addresses social determinants of health and postnatal well-being among women with type 2 diabetes in Thailand; however, several aspects require clarification to improve coherence and alignment with the study.

Comment 5:

Regarding the structure and flow of ideas, the progression is not clearly organized. The text introduces SDoH in general terms (lines 58–66), then moves to diabetes in pregnancy (lines 68–75), and later to postpartum challenges (lines 77–82), but the connections between these sections are not explicitly developed. In addition, postpartum challenges and care needs are described more than once (lines 77–82 and again lines 97–103), creating some repetition without adding new elements. As a result, the research gap is not built in a clear and progressive way. The introduction would benefit from a clearer progression that consistently builds toward the study rationale.

Comment 6:

Regarding the study aim and objectives, the manuscript first states a general aim (lines 90–92) and later presents four specific aims (lines 105–111) . It is not clear whether the first statement represents the main aim or a summary of the four aims. In addition, Aim 1 (translation of the questionnaire) appears to be a methodological or preparatory step

rather than directly aligned with the stated aim of describing social determinants of health and postnatal well-being. In contrast, Aims 2–4 address substantive aspects of the research question but extend beyond description by including exploration of women's experiences and analysis of postnatal care. The manuscript does not clearly explain whether Aim 1 is part of the scientific objective or a step required to conduct the study, nor how Aims 3 and 4 relate to the overall aim. It would be helpful to present the general aim and the specific aims together in a single section and clearly indicate how each specific aim contributes to the overall purpose of the study.

Comment 7:

Regarding the alignment between the introduction and the study components, the introduction emphasizes understanding SDoH and women's postnatal experiences, but it does not explain why the Postnatal Well-being in Transition questionnaire is required to address this objective. The instrument is introduced later in the aims without prior justification. It is therefore unclear what this tool specifically measures in relation to SDoH and postnatal well-being, and why it is necessary for this study. Please clarify the role and relevance of this instrument in relation to the research problem.

Comment 8:

Regarding the mixed-methods design, its usefulness for this study is generally understandable, as the study combines measurement of postnatal well-being/SDoH with exploration of women's experiences. However, this rationale is not clearly stated in the introduction. The manuscript explains the design and its procedures, but the justification for why this approach is the most appropriate for the research problem is underdeveloped. This point would benefit from clearer and more explicit wording in the introduction.

Comment 9:

Regarding the focus on type 2 diabetes (T2DM), the introduction states that T2DM affects pregnancy but does not explain why this condition is specifically selected instead of gestational diabetes or other types. This is particularly relevant given that the study population includes women aged 20–44 years, while T2DM is generally more prevalent at older ages. The manuscript does not provide epidemiological or contextual justification for this choice. Please clarify the rationale for focusing on T2DM in this population.

Comment 10:

It is unclear why both the flow chart and the table are needed, as they appear to present the same information in different formats. Even if the authors choose to retain both, their

placement in the introduction is not well justified, as this type of content would be more appropriately located in the methods section.

Summary of recommendation for the introduction

Overall, the introduction would benefit from (1) reorganizing the sequence of ideas to clearly build the research gap and study rationale; (2) removing repeated statements and redundancies, particularly in the description of postpartum challenges and care needs; (3) presenting the aim and specific objectives in a consistent and coherent structure and in a single location; (4) clearly aligning the background with the study scope, the use of the Postnatal Well-being in Transition instrument, and the mixed-methods design, with explicit justification of these choices; (5) providing a clear and explicit rationale for the selection of T2DM as the study population, including its relevance to this age group and context; and (6) improving the precision of key statements and ensuring appropriate alignment between claims and supporting references, prioritizing direct empirical or primary sources over secondary or conceptual citations where aplicable; and 7) Consider removing one of the two elements to avoid redundancy and relocate the remaining figure/table to the methods section, where it more appropriately supports the study design description.

B) METHOD

<u>Comment 11 (Overall structure and organization, lines 161–311):</u>

The Methods section (lines 161–311) presents all key components of the study; however, its structure and organization are difficult to follow. Subsections are fragmented and sometimes repetitive (e.g., "Quantitative phase," "Quantitative data collection," and "Quantitative Phase" reappear, with similar repetition for qualitative components), which disrupts the logical flow. Some information, such as recruitment procedures and data collection approaches, is introduced more than once, while other elements are separated across different sections, making it difficult to understand the full study process as a coherent sequence. The Methods would benefit from being reorganized into a clearer and more conventional structure, where each component is described once and in a logical order.

<u>Comment 12 (Reporting of mixed-methods design):</u>

The reporting of the mixed-methods design would benefit from alignment with established reporting and appraisal frameworks for mixed-methods research. In particular, the manuscript could be strengthened by following the Good Reporting of A Mixed Methods Study (GRAMMS) guidelines, which emphasize clear justification for the use of

mixed methods, explicit description of the study design, and transparent reporting of how quantitative and qualitative components are integrated. This is especially relevant given the current limitations in the clarity of the rationale for the mixed-methods approach and the description of integration between phases.

In addition, consideration of established appraisal tools such as the Mixed Methods Appraisal Tool (MMAT) may help ensure coherence between the research questions, study design, data collection, and integration of findings. Using these frameworks as guidance would improve the transparency, completeness, and methodological rigor of the protocol and facilitate its evaluation by readers.

Here there are some references that could be of some values for the authors

Good Reporting of A Mixed Methods Study (GRAMMS)

- O'Cathain A, Murphy E, Nicholl J. The quality of mixed methods studies in health services research. J Health Serv Res Policy. 2008;13(2):92–98. doi: 10.1258/jhsrp.2007.007074.
- Munce, Sarah et al. "Updating the Good Reporting of a Mixed Methods Study (GRAMMS) Reporting Guidelines: Protocol for a Methodological Review and Modified Delphi Process." JMIR research protocols vol. 15 e82364. 17 Mar. 2026, doi:10.2196/82364

Mixed Methods Appraisal Tool (MMAT) Version 2018 User Guide

- Hong, Q.N., Pluye, P., Fàbregues, S., Bartlett, G., Boardman, F., Cargo, M., et al. (2018). Mixed Methods Appraisal Tool (MMAT), version 2018. Registration of Copyright (#1148552), Canadian Intellectual Property Office, Industry Canada. https://www.nccmt.ca/knowledge-repositories/search/232

Comment 13 (Study design, lines 137–142):

The study design is partially explained in the Introduction, which creates some redundancy. It would be more appropriate to present the rationale for using an explanatory sequential mixed-methods design in the Introduction, and reserve the Methods section for a clear and specific description of how the design is implemented in this study.

Comment 14 (Research setting, lines 143–149):

The description of the research setting is too general. The manuscript states that the study will be conducted in two public hospitals (urban and suburban), but the specific locations

and relevant characteristics of these settings are not provided. More detailed information is needed to justify their selection and to allow readers to assess the context and potential applicability of the findings.

In addition, the statement that the study aims to achieve “maximum variation” is not fully supported by the proposed sampling strategy. With a total of 50 participants for the quantitative phase and 12 for the qualitative phase, recruited from only two sites, it is unlikely that maximum variation will be achieved. While some degree of variation is expected and desirable, the term “maximum variation” may be overstated in this context and should be reconsidered or more carefully justified.

Comment 15 (Study phases and consistency, lines 148–149):

The statement that the study is divided into two phases, (1) translation of the instrument and (2) data collection for the mixed-methods study, is not fully consistent with the way the study is presented in Figure 1 and Table 1 . In these, the translation process appears as a component within the quantitative phase rather than as a separate phase. This creates some inconsistency in how the study design is conceptualized and presented. Please ensure that the description of study phases is clear, consistent, and aligned across the text, figures, and tables.

Comment 16 (Translation and validation of the instrument):

The description of the translation and validation process of the Postnatal Well-being in Transition questionnaire (lines 151–159) is unclear and uses imprecise terminology, which makes it difficult to assess the methodological rigor of this component.

First, the term “translation tool” is not appropriate; it would be more accurate to refer to this as “translation and cross-cultural adaptation of the instrument.” In addition, while the manuscript states that the Beaton et al. six-stage process was followed, the steps are not clearly described or mapped to the standard components of this methodology (e.g., forward translation, synthesis, back-translation, expert committee review, pre-testing).

Second, the description of back-translation (“to ensure clarity and cultural appropriateness”) is conceptually inaccurate. Back-translation is primarily used to assess equivalence with the original version, not directly to ensure cultural appropriateness, which is typically addressed through expert review and pre-testing.

Third, the validation process is insufficiently described. The manuscript states that three experts validated the questionnaire and that the content validity index (CVI) equals 1; however, it is unclear how this was calculated (e.g., I-CVI vs. S-CVI), what criteria were

used, and whether this reflects item-level or scale-level validity. Reporting a CVI of 1 without further detail raises concerns about the robustness of the validation process.

Finally, the statement that the CVI “will be calculated before implementation” is inconsistent with the subsequent statement that it “equals 1,” suggesting a lack of clarity about whether this step has already been completed or is planned.

Recommendation: Please revise this section to use appropriate methodological terminology, clearly describe each step of the translation and cross-cultural adaptation process according to established guidelines, and provide a transparent and consistent description of the validation procedures, including how the CVI was calculated and interpreted. In addition, please clarify whether the translation and validation processes have already been completed or are planned. If these steps have already been carried out, please justify why the original and translated versions of the instrument, as well as the results of the validation procedures, are not included as supplementary material.

Comment 17 (Study timeline and protocol status, lines 171–173):

The manuscript states that recruitment and data collection will occur from September 2025 to December 2026 ; however, given that 2025 has already passed, this raises uncertainty about the current status of the study. In addition, other parts of the manuscript suggest that some steps may already have been conducted (e.g., content validity index reported as 1, translation described in past tense). It is therefore unclear whether the study is planned, ongoing, or partially completed. Please clarify the current status of the study and ensure that the timeline and verb tense are consistent throughout the Methods section.

Comment 18 (Sampling strategy, lines 182–207):

The description of sampling in the qualitative component is conceptually unclear and suggests inconsistency in the use of methodological terminology. The manuscript refers to multiple sampling approaches (purposeful, nested, stratified, and theoretical sampling), but it is not clearly specified which strategy is actually being implemented. The practical procedure described—selection of participants based on high and low scores—corresponds to a form of purposeful sampling (e.g., extreme case selection), yet this is not explicitly stated. Instead, the text combines definitions of different sampling strategies without clearly explaining how participants will be selected in practice, creating a mismatch between terminology and implementation. The sampling strategy should be simplified and clearly described, focusing on what is actually being done.

Comment 19 (Qualitative sample size and saturation, lines 187–189):

The manuscript justifies the qualitative sample size (n=12) based on a previous study in which saturation was reached at 12 participants; however, this reflects a misunderstanding of saturation. In qualitative research, saturation refers to the point during data analysis at which additional interviews or observations no longer yield new themes, insights, or relevant information, indicating that further data collection may not be necessary. It is therefore a process that emerges during analysis rather than a fixed number that can be determined in advance or transferred from previous studies, as it depends on the complexity of the research question and the richness of the data. Presenting saturation as predetermined may weaken the methodological rigor of the qualitative component. The sample size should instead be described as an initial estimate, with clarification that the final number of participants will be guided by saturation during analysis, or justified using an alternative rationale appropriate for qualitative research.

Comment 20 (Quantitative sample size, lines 174–176):

The quantitative sample size (n=50) is cited from a previous study (Maghalian et al., 2024) , but no explanation is provided regarding its adequacy for the objectives of this study. It is unclear whether this number is based on feasibility, prior studies, or other considerations. A clearer and more explicit rationale for the quantitative sample size is needed.

Comment 21 (Redundancy in data collection and analysis sections, lines 174–231 and 237–251):

Descriptions of quantitative and qualitative data collection and analysis are fragmented across multiple subsections, with some elements repeated in different parts of the Methods. For example, recruitment and procedures are described more than once, which creates redundancy and makes it difficult to follow the sequence of study activities. Consolidating all quantitative procedures into a single subsection and all qualitative procedures into another would improve clarity and reduce repetition.

Comment 22 (Instruments and measurement of SDoH, lines 219–231):

The instruments used in the study are identifiable; however, their characteristics and measurement properties are insufficiently described. The Postnatal Well-being in Transition questionnaire is a previously developed and published instrument, but the manuscript does not provide sufficient information on its key properties (e.g., structure, domains, or prior validation) to assess its appropriateness for this study population and context, particularly in relation to its translation and adaptation. In contrast, the description of the SDoH measurement is limited. The manuscript indicates that the questionnaire is “based on previous research” and reports a content validity index of 1

from three experts, but it is not clear whether this represents an existing validated instrument, an adaptation, or a newly developed set of items. There is also insufficient detail on the structure of the questionnaire, how variables are operationalized, and how the CVI was calculated. This level of reporting is not sufficient to assess the validity and appropriateness of the measure.

Comment 23 (Mixed-methods integration and triangulation, lines 274–282):

The manuscript describes triangulation and integration of qualitative and quantitative findings; however, this section largely repeats general methodological concepts already presented earlier (e.g., in the study design and Table 1), rather than focusing on how integration will be specifically implemented in this study. This contributes to redundancy without improving clarity.

Comment 24 (In-text citation style and adherence to PLOS guidelines):

In several parts of the Methods section, in-text citations do not appear to follow PLOS citation guidelines and instead resemble an APA-style format (e.g., author name and year), without a corresponding numbered reference that clearly matches the reference list . This creates inconsistency and may affect the traceability of sources. Please ensure that all in-text citations follow PLOS guidelines (numbered citation style) and that each citation corresponds accurately to the reference list.

Summary of recommendation for the method section:

The Methods section would benefit from substantial revision to:

(1) restructure the section to improve logical flow and eliminate repetition, ensuring that each component of the study is presented once and in a clear sequence; if appropriate, the authors may consider following established guidelines for mixed-methods reporting, such as the Good Reporting of A Mixed Methods Study (GRAMMS) or the Mixed Methods Appraisal Tool (MMAT).

(2) clarify the status of the study (planned, ongoing, or partially completed), including consistency between timelines and reported completed steps, and ensure consistent use of verb tense throughout;

(3) streamline the description of the study design to avoid redundancy with the Introduction and clearly distinguish between conceptual rationale and methodological implementation;

(4) improve the description and justification of the research setting, including specifying locations and relevant characteristics, and reconsider claims such as “maximum variation” in light of the actual sampling scope;

(5) simplify and accurately describe the sampling strategy, using appropriate and consistent methodological terminology and clearly explaining how participants are selected in practice;

(6) ensure the correct use and explanation of methodological concepts (e.g., sampling strategies, back-translation), avoiding conceptual inaccuracies and inconsistencies;

(7) provide stronger and more appropriate justification for both quantitative and qualitative sample sizes, avoiding the treatment of saturation as a fixed number and ensuring alignment with qualitative research principles;

(8) describe the characteristics, structure, adaptation, and validation of all instruments—particularly the SDoH measure and the translated questionnaire—in sufficient detail to allow assessment of their appropriateness, including clarification of whether instruments were adapted or newly developed and whether translation/validation has been completed;

(9) ensure that the reported procedures are clearly aligned with established methodological frameworks (e.g., Beaton guidelines), with transparent description of each step;

(10) ensure consistency across the text, figures, and tables (e.g., in the description of study phases);

(11) reduce redundancy in methodological descriptions, particularly regarding data collection, analysis, and integration, and avoid repetition of general methodological concepts;

(12) enhance transparency and reproducibility by considering inclusion of relevant materials (e.g., translated instrument, validation results) as supplementary files; and

(13) revise all in-text citations to comply with PLOS guidelines, ensuring accurate correspondence with the reference list.
