# Supplementary material for: Social determinants of health and postnatal wellbeing among women with type 2 diabetes in Thailand: An explanatory sequential mixed-methods study protocol
Source: PLoS One. 2026 Jul 29;21(7):e0354735. doi: 10.1371/journal.pone.0354735 (PMC13419172; doi:10.1371/journal.pone.0354735)
Supplement: Attachment — Submitted filename: Response to reviewers.docx [file pone.0354735.s005.docx]

**Journal:** PLOS One

**Topic:** Social Determinants of Health and Postnatal Wellbeing among Women with Type 2 Diabetes in Thailand: An Explanatory Sequential Mixed-Methods Study protocol

PONE-D-25-52097

Dear Editor,

We appreciate the opportunity to submit our revised manuscript. Thank you for dedicating your time and effort to providing valuable feedback on our work.

We have addressed the editor's and reviewers’ comments and concerns in a point-by-point manner. Please find our responses to each of them outlined below. We believe the manuscript has been considerably strengthened with these revisions.

| **Editor comments** | **Authors’ Response** |
| --- | --- |
| See comments below. See also attached the materials provided by reviewer(s) whenever they are provided, if helpful. | We thank the reviewers for the thorough evaluation and valuable feedback. We have carefully addressed all concerns in the revised manuscript. |

| **Reviewer #1 comments** | **Authors’ Response** |
| --- | --- |
| Reviewer #1: General evaluation: the author presents an interesting Sequential Explanatory Mixed Methods (QUAN; qual) study addressing women’s health in the Thai context. The integration of REDCap for data management and the hybrid thematic analysis approach are strengths. | Thank you very much for your insightful suggestions. |
| However, there are methodological discrepancies and terminological inconsistencies, particularly regarding instrument validation and sampling logic, that must be addressed to meet the standards of this journal.  1. Content Validity Index (CVI) and Expert Panel Size. The author states that the questionnaires were validated by a panel of three experts. While three is the absolute minimum, it poses a statistical risk. According to Lynn (1986) and Polit & Beck, with a panel of three, the required I-CVI for an item to be considered valid must be 1.00 (100% agreement). Furthermore, if any items were modified based on expert feedback, please describe the iterative process. For a study of this scope, a larger panel (5–10 experts) is preferred to ensure robustness. The protocol lists the "Interview Guide" as being translated and validated via CVI during the initial Quantitative Phase. However, the subsequent section correctly notes that the qualitative probes will be informed by the quantitative results. | Thanks for pointing this out. We retained the panel of three experts, and the revised version of the questionnaires will be reported in the final manuscript. |

| **Reviewer #2 comments** | **Authors’ Response** |
| --- | --- |
| Here I present a summary of recommendations; the full document with detailed comments and recommendations is attached as a **PDF file.**  This manuscript addresses a highly relevant and meaningful topic, focusing on social determinants of health and postnatal well-being among women with type 2 diabetes, an area where further evidence is clearly needed to inform clinical practice and health policy. The study has the potential to contribute valuable insights, particularly given its mixed-methods approach and its focus on a specific population and context. My comments are provided in the spirit of strengthening the manuscript, with particular emphasis on improving clarity of reporting, methodological transparency, and reproducibility. These aspects are especially important in protocol papers, as they allow readers to understand the planned study in detail and to assess any deviations when the final results are published. For clarity, my comments and recommendations are organized into two sections: (1) Introduction and (2) Methods. Each section concludes with a summary of key recommendations to facilitate revision.  **Summary of recommendation for the introduction** Overall, the introduction would benefit from (1) reorganizing the sequence of ideas to clearly build the research gap and study rationale;  (2) removing repeated statements and redundancies, particularly in the description of postpartum challenges and care needs;  (3) presenting the aim and specific objectives in a consistent and coherent structure and in a single location;  (4) clearly aligning the background with the study scope, the use of the Postnatal Well-being in Transition instrument, and the mixed-methods design, with explicit justification of these choices;  (5) providing a clear and explicit rationale for the selection of T2DM as the study population, including its relevance to this age group and context;  6) improving the precision of key statements and ensuring appropriate alignment between claims and supporting references, prioritizing direct empirical or primary sources over secondary or conceptual citations where applicable;  7) Consider removing one of the two elements to avoid redundancy and relocate the remaining figure/table to the methods section, where it more appropriately supports the study design description. | Thank you very much for your helpful feedback. We have carefully addressed all concerns in the revised manuscript. |
| **A) INTRODUCTION**  **Specific comments**  **Comment 1:**  The opening statement (lines 58 and 59) requires clarification, particularly given its  position as the first sentence of the manuscript.  It conflates three distinct components: (1) a shift toward population health/value-based  care, (2) SDOH as a “key strategy,” and (3) achieving health equity as an outcome. It does  not specify where, according to whom, or based on what type of evidence these claims are made, which makes the statement too broad and insufficiently contextualized.  Regarding the supporting citation for this opening statement, the manuscript relies on  reference 1 to support this statement. However, reference 1 is a narrative scientific review reflecting expert consensus, not primary empirical evidence. Moreover, the specific statement cited in reference 1 is not based on direct empirical findings within that paper; instead, it draws on other sources, mainly policy documents (e.g., CMS Equity Plan, HHS Action Plan, Chin 2016), which the authors may wish to consult directly to better align the citation with the intended claim.  As a result, the manuscript is citing a conceptual statement from a review article that itself relies on policy-oriented references, rather than directly citing empirical or primary evidence. This creates a mismatch between the strength of the claim made in the  manuscript and the type of evidence used to support it, and may give the impression that  the statement is more strongly evidence-based than is actually demonstrated by the cited  source.  I recognize that these claims are supported by empirical evidence in the broader literature  and are consistent with current understanding. However, because this is a strong and  important statement, particularly as the opening sentence of the manuscript, it requires greater accuracy and precision in how it is phrased and supported. It would be more appropriate to cite direct empirical or policy-level references that substantiate each  component of the claim, rather than relying indirectly on a conceptual statement from a  narrative review article. | Thank you for your observation. We have added further citation [2] to the manuscript to provide more depth and support for our responses. |
| **Comment 2**  The reference cited for the definition of social determinants of health (lines 60 to 64)  appears to be a website and is not formatted according to PLOS citation guidelines (e.g.,  missing standard elements such as organization, publication details, and access date). The authors should revise this citation to comply with the required format.  Additionally, the authors may consider citing more authoritative or primary sources for this  definition, such as official reports, white papers, or position statements. In particular, the concept of social determinants of health has been more broadly and originally established by the World Health Organization (e.g., WHO Commission on Social Determinants of Health), whereas the Healthy People 2030 framework reflects a U.S.-specific adaptation. Using a more foundational or internationally recognized source may strengthen the conceptual grounding of the manuscript. | Thank you for your observation. We have added further citation [2] to the manuscript to provide more depth and support for our responses. |
| **Comment 3**  In the manuscript, the statement “SDoH can have a greater impact on health outcomes  than healthcare services or personal lifestyle choices alone” (lines 64 to 66) is supported  by reference 3. However, reference 3 is a Commentary-type article that does not present  original empirical data but instead cites other studies to support this claim. While the  underlying evidence exists, the manuscript is relying on a secondary source that  summarizes this evidence, rather than citing the primary empirical studies directly.  In addition, the primary purpose of the cited paper is not to evaluate or demonstrate this  claim, but to discuss the need for data science methods and capacity in the study of SDoH. Therefore, the reference is not fully aligned with the specific statement it is being used to  support.  For a statement of this strength, it would be more appropriate to cite the original empirical  studies that provide the supporting evidence and are more directly aligned with the claim. | Thank you for your observation. We have added further citation [5] to the manuscript to provide more depth and support for our responses. |
| **Comment 4**  The statement “Additionally, in the early postpartum period, those women may struggle to breastfeed while maintaining reasonably stable blood glucose levels” (lines 81 and 82) is supported by reference 10; however, this paper primarily focuses on the validation of a  questionnaire (DBM-Q) rather than providing direct empirical evidence quantifying this  specific difficulty. While the introduction acknowledges that women may face challenges managing breastfeeding and glycaemic variability, the study itself is not designed to substantively evaluate or demonstrate this claim. Therefore, the reference only provides indirect contextual support, and a more appropriate citation would be a study specifically investigating breastfeeding-related glycaemic instability in the early postpartum period. | Thank you for your observation. We have added further citation [13] to the manuscript to provide more depth and support for our responses. |
| **General comments**  The introduction addresses social determinants of health and postnatal well-being among women with type 2 diabetes in Thailand; however, several aspects require clarification to improve coherence and alignment with the study.  **Comment 5:**  Regarding the structure and flow of ideas, the progression is not clearly organized. The  text introduces SDoH in general terms (lines 58–66), then moves to diabetes in pregnancy  (lines 68–75), and later to postpartum challenges (lines 77–82), but the connections  between these sections are not explicitly developed. In addition, postpartum challenges  and care needs are described more than once (lines 77–82 and again lines 97–103),  creating some repetition without adding new elements. As a result, the research gap is not  built in a clear and progressive way. The introduction would benefit from a clearer  progression that consistently builds toward the study rationale. | Thank you for your suggestion. |
| **Comment 6:**  Regarding the study aim and objectives, the manuscript first states a general aim (lines 90–92) and later presents four specific aims (lines 105–111). It is not clear whether the first statement represents the main aim or a summary of the four aims. In addition, Aim 1  (translation of the questionnaire) appears to be a methodological or preparatory step rather than directly aligned with the stated aim of describing social determinants of health  and postnatal well-being. In contrast, Aims 2–4 address substantive aspects of the  research question but extend beyond description by including exploration of women’s experiences and analysis of postnatal care. The manuscript does not clearly explain whether Aim 1 is part of the scientific objective or a step required to conduct the study, nor how Aims 3 and 4 relate to the overall aim. It would be helpful to present the general aim and the specific aims together in a single section and clearly indicate how each specific aim contributes to the overall purpose of the study. | We have decided to maintain the research aims as originally stated, as they represent the chronological steps of our study process. This sequence begins with the instrument translation and validation (Aim 1), followed by the exploratory sequential mixed-methods phases, where Aims 2, 3, and 4 represent the quantitative, qualitative, and mixed-methods components, respectively. |
| **Comment 7:**  Regarding the alignment between the introduction and the study components, the  introduction emphasizes understanding SDoH and women’s postnatal experiences, but it  does not explain why the Postnatal Well-being in Transition questionnaire is required to address this objective. The instrument is introduced later in the aims without prior  justification. It is therefore unclear what this tool specifically measures in relation to SDoH  and postnatal well-being, and why it is necessary for this study. Please clarify the role and relevance of this instrument in relation to the research problem. | We have structured the manuscript by outlining four research aims, followed by their respective quantitative, qualitative, and mixed-methods approaches. Specific measures and data collection strategies are detailed for each variable accordingly. |
| **Comment 8:**  Regarding the mixed-methods design, its usefulness for this study is generally  understandable, as the study combines measurement of postnatal well-being/SDoH with exploration of women’s experiences. However, this rationale is not clearly stated in the introduction. The manuscript explains the design and its procedures, but the justification  for why this approach is the most appropriate for the research problem is underdeveloped. This point would benefit from clearer and more explicit wording in the  introduction. | Thank you! |
| **Comment 9:**  Regarding the focus on type 2 diabetes (T2DM), the introduction states that T2DM affects pregnancy but does not explain why this condition is specifically selected instead of gestational diabetes or other types. This is particularly relevant given that the study  population includes women aged 20–44 years, while T2DM is generally more prevalent at  older ages. The manuscript does not provide epidemiological or contextual justification for  this choice. Please clarify the rationale for focusing on T2DM in this population. | We have justified the knowledge gap in the Introduction as follows: “To date, no study has explored how women with type 2 diabetes experience life after childbirth. This study aims to describe the social determinants of health and postnatal well-being during the transition for postpartum women with T2DM.” (See Page 3, Lines 89–92) |
| **Comment 10:**  It is unclear why both the flow chart and the table are needed, as they appear to present  the same information in different formats. Even if the authors choose to retain both, their  placement in the introduction is not well justified, as this type of content would be more appropriately located in the methods section. | We have decided to retain both the flowchart and the table in the manuscript to provide a comprehensive overview of the study process. |
| **Summary of recommendation for the introduction**  Overall, the introduction would benefit from (1) reorganizing the sequence of ideas to  clearly build the research gap and study rationale; (2) removing repeated statements and redundancies, particularly in the description of postpartum challenges and care needs; (3) presenting the aim and specific objectives in a consistent and coherent structure and in a single location; (4) clearly aligning the background with the study scope, the use of the Postnatal Well-being in Transition instrument, and the mixed-methods design, with explicit justification of these choices; (5) providing a clear and explicit rationale for the selection of T2DM as the study population, including its relevance to this age group and context; and (6) improving the precision of key statements and ensuring appropriate alignment between claims and supporting references, prioritizing direct empirical or primary sources over secondary or conceptual citations where applicable; and 7) Consider removing one of the two elements to avoid redundancy and relocate the remaining figure/table to the methods section, where it more appropriately supports the study design description. | We have made amendments to our manuscript. Please refer to the revised version. |
| **B) METHOD**  Comment 11 (Overall structure and organization, lines 161–311):  The Methods section (lines 161–311) presents all key components of the study; however,  its structure and organization are difficult to follow. Subsections are fragmented and  sometimes repetitive (e.g., “Quantitative phase,” “Quantitative data collection,” and  “Quantitative Phase” reappear, with similar repetition for qualitative components), which  disrupts the logical flow. Some information, such as recruitment procedures and data  collection approaches, is introduced more than once, while other elements are separated  across different sections, making it difficult to understand the full study process as a  coherent sequence. The Methods would benefit from being reorganized into a clearer and more conventional structure, where each component is described once and in a logical  order. | We added more information about recruitment process and revise overall flow: “Potential participants will be identified at urban and suburban hospital sites through medication records and clinical visits during the postnatal period. Interested individuals will be directed to a secure online platform (Research Electronic Data Capture: REDCap), where they will view the online informed consent form. Participants must pass a brief 'consent check' question before the system allows them to proceed to the survey, ensuring they have read and understood the study requirements. We will monitor non-response rates by tracking the number of individuals who do not wish to complete the survey. Additionally, mode equivalence will be monitored to ensure that data collection remains consistent across both urban and suburban settings.” See Page 7 Lines 219-227. |
| Comment 12 (Reporting of mixed-methods design):  The reporting of the mixed-methods design would benefit from alignment with  established reporting and appraisal frameworks for mixed-methods research. In particular, the manuscript could be strengthened by following the Good Reporting of A Mixed Methods Study (GRAMMS) guidelines, which emphasize clear justification for the use of mixed methods, explicit description of the study design, and transparent reporting of how  quantitative and qualitative components are integrated. This is especially relevant given  the current limitations in the clarity of the rationale for the mixed-methods approach and  the description of integration between phases.  In addition, consideration of established appraisal tools such as the Mixed Methods  Appraisal Tool (MMAT) may help ensure coherence between the research questions, study design, data collection, and integration of findings. Using these frameworks as guidance would improve the transparency, completeness, and methodological rigor of the protocol and facilitate its evaluation by readers.  Here there are some references that could be of some values for the authors  Good Reporting of A Mixed Methods Study (GRAMMS)  • O'Cathain A, Murphy E, Nicholl J. The quality of mixed methods studies in health  services research. J Health Serv Res Policy. 2008;13(2):92–98. doi:  10.1258/jhsrp.2007.007074.  • Munce, Sarah et al. “Updating the Good Reporting of a Mixed Methods Study  (GRAMMS) Reporting Guidelines: Protocol for a Methodological Review and  Modified Delphi Process.” JMIR research protocols vol. 15 e82364. 17 Mar. 2026,  doi:10.2196/82364  Mixed Methods Appraisal Tool (MMAT) Version 2018 User Guide  • Hong, Q.N., Pluye, P., Fàbregues, S., Bartlett, G., Boardman, F., Cargo, M., et al.  (2018). Mixed Methods Appraisal Tool (MMAT), version 2018. Registration of  Copyright (#1148552), Canadian Intellectual Property Office, Industry Canada.  https://www.nccmt.ca/knowledge-repositories/search/232 | Thank you very much for your suggestions. |
| Comment 13 (Study design, lines 137–142):  The study design is partially explained in the Introduction, which creates some  redundancy. It would be more appropriate to present the rationale for using an  explanatory sequential mixed-methods design in the Introduction, and reserve the  Methods section for a clear and specific description of how the design is implemented in this study. | Thank you! |
| Comment 14 (Research setting, lines 143–149):  The description of the research setting is too general. The manuscript states that the study  will be conducted in two public hospitals (urban and suburban), but the specific locations and relevant characteristics of these settings are not provided. More detailed information is needed to justify their selection and to allow readers to assess the context and potential applicability of the findings.  In addition, the statement that the study aims to achieve “maximum variation” is not fully  supported by the proposed sampling strategy. With a total of 50 participants for the  quantitative phase and 12 for the qualitative phase, recruited from only two sites, it is  unlikely that maximum variation will be achieved. While some degree of variation is  expected and desirable, the term “maximum variation” may be overstated in this context  and should be reconsidered or more carefully justified. | We added more details: “This study will be conducted across two public hospitals in Thailand—one urban and one suburban. The urban site is a tertiary care center in Bangkok, serving a high-density population with diverse socioeconomic backgrounds, ranging from low to high income. In contrast, the suburban hospital in Samut Prakan serves a more stable, residential, and primarily middle-income community. This dual-site approach utilizes maximum variation sampling to capture a diverse range of experiences. By selecting sites with distinct geographical and socioeconomic characteristics, the study aims to account for the varying SDoH that may influence maternal well-being during the postpartum transition.” See Page 6 Lines 161-168. |
| Comment 15 (Study phases and consistency, lines 148–149):  The statement that the study is divided into two phases, (1) translation of the instrument  and (2) data collection for the mixed-methods study, is not fully consistent with the way  the study is presented in Figure 1 and Table 1. In these, the translation process appears as  a component within the quantitative phase rather than as a separate phase. This creates  some inconsistency in how the study design is conceptualized and presented. Please  ensure that the description of study phases is clear, consistent, and aligned across the text,  figures, and tables. | Thank you for your suggestion. |
| Comment 16 (Translation and validation of the instrument):  The description of the translation and validation process of the Postnatal Well-being in Transition questionnaire (lines 151–159) is unclear and uses imprecise terminology, which makes it difficult to assess the methodological rigor of this component.  First, the term “translation tool” is not appropriate; it would be more accurate to refer to this as “translation and cross-cultural adaptation of the instrument.” In addition, while the manuscript states that the Beaton et al. six-stage process was followed, the steps are not clearly described or mapped to the standard components of this methodology (e.g., forward translation, synthesis, back-translation, expert committee review, pre-testing). Second, the description of back-translation (“to ensure clarity and cultural  appropriateness”) is conceptually inaccurate. Back-translation is primarily used to assess  equivalence with the original version, not directly to ensure cultural appropriateness,  which is typically addressed through expert review and pre-testing. Third, the validation process is insufficiently described. The manuscript states that three experts validated the questionnaire and that the content validity index (CVI) equals 1; however, it is unclear how this was calculated (e.g., I-CVI vs. S-CVI), what criteria were used, and whether this reflects item-level or scale-level validity. Reporting a CVI of 1 without further detail raises concerns about the robustness of the validation process.  Finally, the statement that the CVI “will be calculated before implementation” is  inconsistent with the subsequent statement that it “equals 1,” suggesting a lack of clarity  about whether this step has already been completed or is planned.  Recommendation: Please revise this section to use appropriate methodological  terminology, clearly describe each step of the translation and cross-cultural adaptation  process according to established guidelines, and provide a transparent and consistent  description of the validation procedures, including how the CVI was calculated and  interpreted. In addition, please clarify whether the translation and validation processes  have already been completed or are planned. If these steps have already been carried out,  please justify why the original and translated versions of the instrument, as well as the  results of the validation procedures, are not included as supplementary material. | We provided more details “Written authorization to translate, adapt, and validate the *Postnatal Well-being in Transition* questionnaire was granted by the developer of the original tool. We followed the six-stage translation process approach developed by Beaton and colleagues in 2000 [14]. For its use in the Thai population, we culturally translated and adapted the questionnaire into Thai, then back-translate it to ensure clarity and cultural appropriateness. For the forward translation phase, two bilingual Thai-English nursing faculty members independently translated the instrument from English to Thai, with careful consideration given to cultural appropriateness (Forward 1 and Forward 2). The research team then reviewed both versions to produce a synthesized third version (Forward 3). Subsequently, the Forward 3 was sent to two different bilingual Thai-English nursing faculty members for independent back-translation from Thai to English (Back 1 and Back 2). Finally, the research team convened a meeting to resolve any discrepancies and establish the final Thai version of the 'Postnatal Well-being in Transition' scale prior to validation.” See Page 6 Lines173-185. |
| Comment 17 (Study timeline and protocol status, lines 171–173):  The manuscript states that recruitment and data collection will occur from September  2025 to December 2026; however, given that 2025 has already passed, this raises  uncertainty about the current status of the study. In addition, other parts of the  manuscript suggest that some steps may already have been conducted (e.g., content  validity index reported as 1, translation described in past tense). It is therefore unclear  whether the study is planned, ongoing, or partially completed. Please clarify the current  status of the study and ensure that the timeline and verb tense are consistent throughout  the Methods section. | We provided the time line: “The translation of the tool has been completed. Participant recruitment and data collection took place from September 2025 to December 2026. Data collection is now complete, and the results are expected to be finalized by June 2027.” See Page 7 Lines 228-230. |
| Comment 18 (Sampling strategy, lines 182–207):  The description of sampling in the qualitative component is conceptually unclear and  suggests inconsistency in the use of methodological terminology. The manuscript refers to multiple sampling approaches (purposeful, nested, stratified, and theoretical sampling), but it is not clearly specified which strategy is actually being implemented. The practical procedure described—selection of participants based on high and low scores—  corresponds to a form of purposeful sampling (e.g., extreme case selection), yet this is not  explicitly stated. Instead, the text combines definitions of different sampling strategies  without clearly explaining how participants will be selected in practice, creating a  mismatch between terminology and implementation. The sampling strategy should be simplified and clearly described, focusing on what is actually being done. | We have described the sampling strategy in the manuscript on Pages 7–8. |
| Comment 19 (Qualitative sample size and saturation, lines 187–189):  The manuscript justifies the qualitative sample size (n=12) based on a previous study in which saturation was reached at 12 participants; however, this reflects a misunderstanding of saturation. In qualitative research, saturation refers to the point during data analysis at which additional interviews or observations no longer yield new themes, insights, or relevant information, indicating that further data collection may not be necessary. It is therefore a process that emerges during analysis rather than a fixed number that can be determined in advance or transferred from previous studies, as it depends on the complexity of the research question and the richness of the data. Presenting saturation as predetermined may weaken the methodological rigor of the qualitative component. The sample size should instead be described as an initial estimate, with clarification that the  final number of participants will be guided by saturation during analysis, or justified using  an alternative rationale appropriate for qualitative research. Comment 20 (Quantitative sample size, lines 174–176):  The quantitative sample size (n=50) is cited from a previous study (Maghalian et al., 2024), but no explanation is provided regarding its adequacy for the objectives of this study. It is unclear whether this number is based on feasibility, prior studies, or other considerations. A clearer and more explicit rationale for the quantitative sample size is needed. | We have provided an initial estimate for the sample size; however, the final number will be determined based on reaching data saturation.  We added “If data saturation is not reached by the 12th interview, we will continue interviewing participants from both the lower and higher well-being groups accordingly until no new themes emerge.” On Page 7 Lines 248-250. |
| Comment 21 (Redundancy in data collection and analysis sections, lines 174–231 and 237–  251):  Descriptions of quantitative and qualitative data collection and analysis are fragmented  across multiple subsections, with some elements repeated in different parts of the  Methods. For example, recruitment and procedures are described more than once, which creates redundancy and makes it difficult to follow the sequence of study activities. Consolidating all quantitative procedures into a single subsection and all qualitative procedures into another would improve clarity and reduce repetition. | Thank you! |
| Comment 22 (Instruments and measurement of SDoH, lines 219–231):  The instruments used in the study are identifiable; however, their characteristics and  measurement properties are insufficiently described. The Postnatal Well-being in  Transition questionnaire is a previously developed and published instrument, but the  manuscript does not provide sufficient information on its key properties (e.g., structure, domains, or prior validation) to assess its appropriateness for this study population and context, particularly in relation to its translation and adaptation. In contrast, the description of the SDoH measurement is limited. The manuscript indicates that the questionnaire is “based on previous research” and reports a content validity index of 1 from three experts, but it is not clear whether this represents an existing validated instrument, an adaptation, or a newly developed set of items. There is also insufficient detail on the structure of the questionnaire, how variables are operationalized, and how the CVI was calculated. This level of reporting is not sufficient to assess the validity and  appropriateness of the measure. | We have added more details regarding the questionnaire on Pages 8–9. |
| Comment 23 (Mixed-methods integration and triangulation, lines 274–282):  The manuscript describes triangulation and integration of qualitative and quantitative  findings; however, this section largely repeats general methodological concepts already  presented earlier (e.g., in the study design and Table 1), rather than focusing on how  integration will be specifically implemented in this study. This contributes to redundancy  without improving clarity. | Thank you for your feedback. |
| Comment 24 (In-text citation style and adherence to PLOS guidelines):  In several parts of the Methods section, in-text citations do not appear to follow PLOS  citation guidelines and instead resemble an APA-style format (e.g., author name and year), without a corresponding numbered reference that clearly matches the reference list. This creates inconsistency and may affect the traceability of sources. Please ensure that all in-text citations follow PLOS guidelines (numbered citation style) and that each citation corresponds accurately to the reference list. | Thank you for pointing this out. We have double-checked the references. |
| **Summary of recommendation for the method section:**  The Methods section would benefit from substantial revision to:  (1) restructure the section to improve logical flow and eliminate repetition, ensuring that each component of the study is presented once and in a clear sequence; if appropriate, the authors may consider following established guidelines for mixed-methods reporting, such as the Good Reporting of A Mixed Methods Study (GRAMMS) or the Mixed Methods Appraisal Tool (MMAT). | Thank you for your suggestion. |
| (2) clarify the status of the study (planned, ongoing, or partially completed), including consistency between timelines and reported completed steps, and ensure consistent use of verb tense throughout; | We provided the time line: “The translation of the tool has been completed. Participant recruitment and data collection took place from September 2025 to December 2026. Data collection is now complete, and the results are expected to be finalized by June 2027.” See Page 7 Lines 228-230. |
| (3) streamline the description of the study design to avoid redundancy with the Introduction and clearly distinguish between conceptual rationale and methodological implementation; | Thank you! |
| (4) improve the description and justification of the research setting, including specifying locations and relevant characteristics, and reconsider claims such as “maximum variation” in light of the actual sampling scope; | We added more details: “This study will be conducted across two public hospitals in Thailand—one urban and one suburban. The urban site is a tertiary care center in Bangkok, serving a high-density population with diverse socioeconomic backgrounds, ranging from low to high income. In contrast, the suburban hospital in Samut Prakan serves a more stable, residential, and primarily middle-income community. This dual-site approach utilizes maximum variation sampling to capture a diverse range of experiences. By selecting sites with distinct geographical and socioeconomic characteristics, the study aims to account for the varying SDoH that may influence maternal well-being during the postpartum transition.” See Page 6 Lines 161-168. |
| (5) simplify and accurately describe the sampling strategy, using appropriate and consistent methodological terminology and clearly explaining how participants are selected in practice; | We mentioned in our manuscript. See Page 7. |
| (6) ensure the correct use and explanation of methodological concepts (e.g., sampling strategies, back-translation), avoiding conceptual inaccuracies and inconsistencies; | We added the details of translation process. See Page 6. |
| (7) provide stronger and more appropriate justification for both quantitative and qualitative sample sizes, avoiding the treatment of saturation as a fixed number and ensuring alignment with qualitative research principles; | We mentioned in our manuscript. See Page 7. |
| (8) describe the characteristics, structure, adaptation, and validation of all instruments—particularly the SDoH measure and the translated questionnaire—in sufficient detail to allow assessment of their appropriateness, including clarification of whether instruments were adapted or newly developed and whether translation/validation has been completed; | We revised “Second, we will assess the social determinants of health across five domains [2]. The questionnaire for this measure is based on previous research and evidence [4]. This 20-item tool consists of two open-ended questions and 18 frequency-based items designed to quantify women's experiences with social determinants of health (SDoH). There is no formal scoring system for this tool; the responses will be rated as frequency of each item. Validation has been completed; the I-CVI and S-CVI were both 1.0, as determined by a panel of three experts.” See Page 8 Lines 299-303. |
| (9) ensure that the reported procedures are clearly aligned with established methodological frameworks (e.g., Beaton guidelines), with transparent description of each step; | Thank you! We have double-checked the reported procedures. |
| (10) ensure consistency across the text, figures, and tables (e.g., in the description of study phases); | Thanks! |
| (11) reduce redundancy in methodological descriptions, particularly regarding data collection, analysis, and integration, and avoid repetition of general methodological concepts; | Thanks! |
| (12) enhance transparency and reproducibility by considering inclusion of relevant materials (e.g., translated instrument, validation results) as supplementary files; | Thanks! |
| (13) revise all in-text citations to comply with PLOS guidelines, ensuring accurate correspondence with the reference list. | Thanks! |

| **Reviewer #3 comments** | **Authors’ Response** |
| --- | --- |
| Reviewer #3: The authors provided a valid rationale for the propose study. Also the protocol is technically planned, with a visible methodology. | Thank you very much! |

| Reviewer #4 comments | Authors’ Response |
| --- | --- |
| Reviewer #4: Review of Manuscript: Social Determinants of Health and Postnatal Wellbeing among Women with Type 2 Diabetes in Thailand: An Explanatory Sequential Mixed-Methods Study protocol This manuscript presents a protocol for an explanatory sequential mixed-methods study examining the influence of social determinants of health (SDOH) on postnatal wellbeing among women with Type 2 diabetes in Thailand. The study addresses an important and underexplored area at the intersection of maternal health, chronic disease, and social determinants. The mixed-methods design is appropriate to the research question, allowing quantitative assessment followed by qualitative exploration. Overall, the protocol is of potential value. | Thank you for your important comments. |
| I am giving my observations and comments for the author to consider: • Introduction: The abbreviation used is to be consistent. Suggest the author to stick to either SDOH or SDoH | Revised. See Page 1 Line 56. |
| • Line 70 – Does the author mean extensive research exist on SDoH impact on diabetes in pregnancy? | We added “in adults” See Page 1 Line 70. |
| • Line 77-79 - The statement is bit confusing, the difficulties mentioned included delivery but says after childbirth, may suggest to rewrite the statement for clarity | Revised as suggested.  See Page 1 Line 78-79. |
| Quantitative Instrument and Validation • Objectives of the study were clearly stated. | Thank you! |
| • The description of the translation and validation process (Lines 157–159) requires clarification. It should be explicitly stated whether validation has been completed or is planned. | We revised “Validation has been completed; the I-CVI and S-CVI were both 1.0, as determined by a panel of three experts.”  See Page 6 Lines 194-195 |
| • Additional details are needed on the Content Validity Index (CVI), including: o Methods used to calculate item-level (I-CVI) and scale-level (S-CVI) o Criteria for item retention or removal o The process of instrument refinement (e.g., number of items modified or excluded) should be described. | We added “The Item-level Content Validity Index (I-CVI) was calculated as the proportion of experts who gave a rating of 3 (quite relevant) or 4 (very relevant). The Scale-level Content Validity Index (S-CVI), specifically the average scale-level index (S-CVI/Ave), was calculated by summing the I-CVIs of all items and dividing by the total number of items. Any item receiving a score of 1 (not relevant) was considered unacceptable and removed from the scale. Items requiring modification based on expert feedback were revised, and all such adjustments are reflected in the final version of the instrument. Validation has been completed; the I-CVI and S-CVI were both 1.0, as determined by a panel of three experts.” See page 6 Lines173-180. |
| • The manuscript should include a citation for the “Postnatal Wellbeing in Transition” questionnaire | This refers to citation [15], which is already included in the manuscript. |
| Qualitative Sampling and Description • While the linkage between quantitative and qualitative phases is described, the authors should more clearly specify the type(s) of purposive sampling employed (e.g., maximum variation, criterion-based) and the rationale for their use. The paragraph pertaining to it be more refined | We used “This study's qualitative research used purposeful sampling, supported by nested sampling” as mentioned on Page 8 Liens 274-275. |
| Integration of Mixed Methods  • The triangulation is mentioned and described | Thank you! |
| • The integration between quantitative and qualitative findings are explained | Thank you! |
| Minor Comments Ensure consistent terminology throughout (e.g., “postnatal” vs. “postpartum”). | We decided to use the term 'postnatal' and revised the manuscript accordingly. |
| Overall the manuscript would benefit from minor language editing to improve readability. | Thank you! |

| **Reviewer #5 comments** | **Authors’ Response** |
| --- | --- |
| Reviewer #5: This research protocol proposes an explanatory sequential mixed-methods study to explore the social determinants of health (SDoH) and postnatal well-being among women with type 2 diabetes mellitus (T2DM) in Thailand. The study aims to translate the Postnatal Well-being in Transition scale into Thai, quantitatively assess SDoH and well-being, qualitatively explore experiences based on well-being scores, and analyze the impact of postnatal care using a mixed-methods approach. The protocol outlines a two-phase study starting with a quantitative cross-sectional design involving 50 postpartum women, followed by semi-structured interviews with 12 selected participants to deepen the understanding of their transition to motherhood. While the study addresses a critical gap in maternity care and population health, there are substantial methodological and operational details currently missing. | Thank you very much for your suggestions. |
| I would recommend a Major Revision focused on rigorously defining the statistical and operational aspects of the study design, particularly regarding the evaluation of "postnatal care" and the exact linkages between the quantitative and qualitative sampling algorithms. Implementing the suggested revisions would increase the protocol’s reproducibility, transparency, and scientific rigor. | Thank you very much for your suggestions. Please see the revised manuscript for your review. |
| Major Methodological & Statistical Issues 1. Lack of Operationalization for "Postnatal Care" Impact (Aim 4) The protocol states an objective to "explore and analyze the impact of postnatal care on women with type 2 diabetes". [Lines 110-111] **Critique:** The Methods section does not define or measure 'postnatal care' as an exposure or variable, does not describe any comparator groups or a pre/post design, and provides no statistical or qualitative analytic plan intended to estimate or assess impact. Without operationalization of postnatal care and an analytic approach, there is no executable path from the data to an impact estimate. **Recommendation:** The authors must explicitly define how 'postnatal care' will be operationalized and measured. Please specify any comparator groups or exposure levels, and detail the exact statistical or mixed-method analytic approach that will be used to assess its impact. | Thanks for point it out. We revised as “4) evaluate postnatal well-being on women with type 2 diabetes using a mixed-methods approach” See Page 4 Lines 122-123. |
| 2. Incomplete Quantitative Endpoints and Analysis Plan The quantitative phase mentions recruiting 50 participants and performing descriptive statistics [232-235]. **Critique:** The protocol lacks a prespecified quantitative analysis plan, including primary endpoint definitions and statistical tests. **Recommendation:** Specify one or more primary endpoint(s) and the corresponding quantitative analysis and statistical tests. Provide a quantitative sample-size justification (power or precision). Prespecify the quantitative analysis plan for missing data handling, covariates/adjustments, and any intended subgroup or sensitivity analyses. | We added “As this study is descriptive rather than comparative in nature, statistical significance does not drive the determination of target sample size.” on Page 7 Line 232-233.  We also provided “Missing values will be replaced using item mean imputation. Specifically, any missing value for a participant will be substituted with the mean value calculated from all available responses for that variable [19]” See Page 8 Lines 325-327. |
| 3. Ambiguous Qualitative Sampling Algorithm The protocol states that 12 women will be selected for interviews—six with the lowest scores and six with the highest scores from each of six subscales [Lines 183-189]. **Critique:** This description is mathematically ambiguous and contradictory. Selecting six from each of six subscales equals 36 high + 36 low = 72 individuals, which conflicts with the stated N=12 total. Additionally, a clear method for integrating these scores with the qualitative themes is missing. **Recommendation:** Clarify the exact selection rules and the algorithm used to select exactly 12 qualitative interviewees from the quantitative scores, providing accurate sample counts. Specify a reproducible analytic method for linking qualitative themes to participants' mean scores. | For clarification: we used the mean total score rather than individual subscale scores.  “A total of 12 women will be contacted for qualitative interviews: six participants (n=6) with the lowest mean total scores (indicating lower well-being) and six participants (n=6) with the highest mean total scores (indicating higher well-being). Semi-structured phone interviews will be conducted to gain a deeper understanding of postnatal well-being during the transition to motherhood.” See Page 7 Lines 242-246. |
| 4. Superficial Mixed-Methods Integration (Triangulation) The protocol states that a side-by-side joint display will be used to compare and contrast the results for triangulation [Lines 279-283]. **Critique:** Merely presenting qualitative themes next to quantitative statistics constitutes data reporting, not full methodological integration. The protocol lacks a clear explanation of how these two strands will interact analytically. **Recommendation:** Expand on the analytical process for data integration. Beyond placing data side-by-side, please detail the specific procedures for how the qualitative findings will be explicitly used to expand upon, explain, or contextualize the convergence and divergence found in the quantitative data to generate robust meta-inferences. | We revised this section as follows:  “Statistics-by-themes joint displays organize quantitative results (e.g., high and low well-being) across qualitative themes in a matrix format, resembling a crosstabulation. These displays feature qualitative themes in the first column, paired with corresponding statistical data (means and standard deviations). This layout enables the research team to triangulate data, identifying areas of convergence or divergence between the two sources.”  See Page 10 Lines 387-391. |
| 5. Theoretical Framework Congruence and Missing SDoH Tool Justification The protocol bases its SDoH domains on Healthy People 2030. [Introduction section and Lines 105 - 121] **Critique:** Healthy People 2030 is a US-centric framework. The manuscript currently lacks the necessary bibliography or methodological description demonstrating that the specific SDoH instrument chosen has been validated or culturally adapted for use in Thailand. While Objective 1 explicitly details the translation and adaptation of the Postnatal Well-being in Transition scale, similar reporting is absent for the SDoH tool. **Recommendation:** Please provide appropriate citations demonstrating the validity and reliability of the chosen SDoH questionnaire within the Thai population. If the tool has not been previously validated in this context, clarify whether a cultural adaptation process will be conducted, or justify methodologically why the US-centric domains are directly applicable to the Thai socio-cultural and healthcare setting without modification. | We developed the SDoH questionnaire based on Healthy People 2030 [2] and our previous research [4]. This tool covers all five domains and has a Content Validity Index (CVI) of 1.0; it was validated by Thai experts who also evaluated the tool's cultural appropriateness. |
| 6. Qualitative Rigor: Positionality and Reflexivity The protocol details the interview process but omits researcher details [Lines 182 - 212]. **Critique:** Interviewer bias and power dynamics play a critical role in data quality. If healthcare personnel from the participants' own hospital settings conduct the interviews, patients might not provide honest assessments of their well-being or care due to social desirability bias or fear of negative repercussions on their clinical treatment. **Recommendation:** Please include a reflexivity statement addressing the researchers' positionality (specifying the professional background and clinical role of the interviewers). Detail the specific strategies employed to mitigate power dynamics between the interviewer and the participants. | We added “The interviewer is a female nursing faculty member with 12 years of clinical nursing practice and expertise in qualitative data collection. At the start of each interview, she explicitly states that she is acting in a research capacity, rather than a clinical one, to encourage honest disclosure. Interviews are conducted over the phone at times most convenient for the participants. A semi-structured interview guide is used to allow participants to lead the narrative, ensuring their lived experiences take precedence over the researcher's clinical assumptions. The interviewer records field notes regarding the conversational tone, date, and time of each interview.” See Page 9 Lines 331-338. |
| Specific & Minor Comments * Recruitment & Bias: Clarify the recruitment and enrollment workflow, including online consent procedures and site-specific recruitment targets across the urban and suburban hospitals. Describe concrete bias-mitigation measures (e.g., consecutive or stratified recruitment, mode equivalence checks, monitoring nonresponse) and explain how the representativeness of the sample will be assessed. | We provided details “Potential participants will be identified at urban and suburban hospital sites through medication records and clinical visits during the postnatal period. Interested individuals will be directed to a secure online platform (Research Electronic Data Capture: REDCap), where they will view the online informed consent form. Participants must pass a brief 'consent check' question before the system allows them to proceed to the survey, ensuring they have read and understood the study requirements. We will monitor non-response rates by tracking the number of individuals who do not wish to complete the survey. Additionally, mode equivalence will be monitored to ensure that data collection remains consistent across both urban and suburban settings.” See Pages 7 Lines 219-227. |
| * Translation Procedures: Report the qualifications of the forward and back translators and summarize any pilot testing procedures and results used to finalize the Thai version. | Thanks for your comment. We provided additional details here: “For the forward translation phase, two bilingual Thai-English nursing faculty members independently translated the instrument from English to Thai, with careful consideration given to cultural appropriateness (Forward 1 and Forward 2). The research team then reviewed both versions to produce a synthesized third version (Forward 3). Subsequently, the Forward 3 was sent to two different bilingual Thai-English nursing faculty members for independent back-translation from Thai to English (Back 1 and Back 2). Finally, the research team convened a meeting to resolve any discrepancies and establish the final Thai version of the 'Postnatal Well-being in Transition' scale prior to validation.” See Page 6 Lines177-185. |
| * SDoH Instrumentation: Provide the full SDoH instrument items or exact references, and specifically state how each SDoH domain and the postnatal well-being scale will be scored and operationalized for the planned descriptive analyses. | We added “This 20-item tool consists of two open-ended questions and 18 frequency-based items designed to quantify women's experiences with social determinants of health (SDoH). There is no formal scoring system for this tool; the responses will be rated as frequency of each item. Validation has been completed; the I-CVI and S-CVI were both 1.0, as determined by a panel of three experts.” On Page 8 Lines 299-303. |

| **Reviewer #6 comments** | **Authors’ Response** |
| --- | --- |
| 1. Line 59: A typo in “social determinants of health (SDOH)”, should be “SDoH”. | Revised as suggested. |
| 2. Part 2 Study data collection: Will information of delivery mode (C-section, virginal, etc.), blood glucose during pregnancy and postpartum period, medication data, and baby information (gestational age, birth weight, etc.) be collected or be available through medical records? | Data will be collected from mothers when they respond to questionnaires via REDCap. |
| 3. Lines 200-205: The authors mentioned that based on the quantitative phase results, stratified sampling approach will be used to select samples for qualitative interview. It is vague and unclear what subgroups will be here. In the first paragraph of this section, they mentioned that 6 participants with the lowest scores and highest scores will be interviewed, how this will be incorporated in the potentially stratified sampling approach. | Participants will be categorized into subgroups according to their well-being scores. To ensure a broad range of perspectives, we will recruit 12 participants in total—six each from the highest and lowest scoring quartiles of the *Postnatal Well-being in Transition* questionnaire. This approach ensures that extreme experiences are represented within the sample. |
| 4. Lines 219-221: The authors mentioned that they will assess SDoH covering 5 domains and stated that they will apply for questionnaires from previous research with ref. [4]. Ref [4] is a review article on racial and ethnic disparities in health care and health outcomes for pregnancy women with diabetes, it cannot cover all the 5 domains. I would appreciate it if the authors can list the validated questionnaires of SDoH that they plan to use in their forth coming survey. | The SDoH tool guided by Healthy People 2030 and our previous research. This tool covers all five domains and has a Content Validity Index (CVI) of 1.0. |
| 5. Lines 275-278: The authors mentioned that “to conduct triangulation, qualitative and quantitative analyses will be conducted separately”, but it seems that the team adapted a sequential approach that the qualitative samples will be selected based on quantitative scores, then how to make sure that the triangulation process is not bias because this nested sampling strategy? | We thank the reviewer for this insightful comment. We would like to clarify our approach to maintaining the rigor of the triangulation process: 1. We added “Since the sampling is sequential, using quantitative scores to identify participants, the analysis of the qualitative interviews will be conducted independently. Coders will be blinded to the specific individual quantitative scores of the participants during the coding phase.” See Page 9 Lines 341-343. 2. We added “The use of a nested approach is intentional, as this inter-relationship is a recognized strength of the explanatory sequential design; it allows for a deeper understanding of the extreme cases identified in the first phase.” See Page 8 Lines 271-273. 3. We provided more details “To ensure unbiased triangulation, a side-by-side comparison will be conducted during the final integration phase. We will identify exemplary quotations where qualitative themes either converge with or contradict quantitative trends.” See Page 10 Line 384-386. |
| 6. In the data management section, please provide data sharing statement on how will the collected data be published or will data be available upon request. | Thank you for this suggestion. We have added a data availability statement to the data management section on Page 10 Lines 376-379.  “The findings of this study will be published in an international peer-reviewed journal. To protect participant confidentiality and privacy, the de-identified datasets generated during the study are not publicly archived but are available from the corresponding author upon reasonable request.” |

We appreciate your thoughtful comments and suggestions. We believe that through the revision process; the manuscript has been improved. Thank you in advance for your consideration.

We look forward to hearing from you.

Sincerely,

Authors
