# Supplementary material for: Social determinants of health and postnatal wellbeing among women with type 2 diabetes in Thailand: An explanatory sequential mixed-methods study protocol
Source: PLoS One. 2026 Jul 29;21(7):e0354735. doi: 10.1371/journal.pone.0354735 (PMC13419172; doi:10.1371/journal.pone.0354735)
Supplement: Attachment — Submitted filename: Response to reviewers R2.docx [file pone.0354735.s006.docx]

**Journal:** PLOS One

**Topic:** Social Determinants of Health and Postnatal Wellbeing among Women with Type 2 Diabetes in Thailand: An Explanatory Sequential Mixed-Methods Study protocol

PONE-D-25-52097R1

Dear Editor,

We appreciate the opportunity to submit our revised manuscript. Thank you for dedicating your time and effort to providing valuable feedback on our work.

We have addressed the editor's and reviewers’ comments and concerns in a point-by-point manner. Please find our responses to each of them outlined below. We believe the manuscript has been considerably strengthened with these revisions.

| **Journal Requirements** | **Authors’ Response** |
| --- | --- |
| 1. If the reviewer comments include a recommendation to cite specific previously published works, please review and evaluate these publications to determine whether they are relevant and should be cited. There is no requirement to cite these works unless the editor has indicated otherwise. | Thank you very much for your insightful suggestions. |

| **Reviewer #1 comments** | **Authors’ Response** |
| --- | --- |
| The authors have addressed all the comments and concerns raised in the previous review round. The paper is now clear, scientifically sound. I have no further comments. | Thank you very much! |

| **Reviewer #4 comments** | **Authors’ Response** |
| --- | --- |
| The authors have adequately addressed most of the comments raised and the manuscript has improved substantially in terms of detailing and clarity. However there are few minor issues to consider: | Thank you very much! We have carefully addressed all concerns in the revised manuscript. |
| Exclusion criteria: it is not clear whether the clinical status or outcome of the baby (e.g., neonatal complications, NICU admission, stillbirth, neonatal death, congenital anomalies, etc.) will influence participant selection. This may be explicitly stated. | We added “Participants will be eligible for inclusion regardless of infant clinical status (including, but not limited to, NICU admission, congenital anomalies, stillbirth, or neonatal death).” On page 7 lines 190-192. |
| The study objectives and the research phases are still not clearly aligned as previously commented by the reviewers. The translation of the instrument which was pointed out earlier by a reviewer as a methodological step rather than a study objective and may be included as part of the quantitative phase is still listed as the first aim. | We have already listed this as “(1) to culturally adapt and translate the Postnatal Well-being in Transition instrument into Thai” on page 4 lines 115-116. |
| Appreciate that the domains included in the questionnaire are described. However information regarding the number of questions/items under each domain is missing. | We have added item numbers under each domain. Please see the revision on page 9, lines 274-281. |
| The manuscript mentions that data collection will continue until December 2026; however, the study is simultaneously described as completed in the following sentence. Please clarify | We added “Translation of the tool has been completed. Participant recruitment and data collection commenced in September 2025. We expect to complete data collection by December 2026, and the results are projected to be finalized by June 2027.” See page 7 lines 207-209. |
| In Table 1: The Interview guide may be moved to as part of Qualitative phase | Revised as suggested. |

| **Reviewer #5 comments** | **Authors’ Response** |
| --- | --- |
| I appreciate the authors' efforts in revising the manuscript. Several improvements are noted, including the clarification of the qualitative sampling algorithm and the expanded recruitment description. However, three critical concerns remain unresolved. | Thank you very much for your helpful feedback. We have carefully addressed all concerns in the revised manuscript. |
| 1. Objective lacks analytical operationalization Reformulating the objective from "evaluate impact" to "evaluate postnatal well-being using a mixed-methods approach" does not resolve the original concern. Insufficient description of the quantitative-to-qualitative transition | Thanks for pointing that out. Please see a revision on page 11 |
| The quantitative analysis plan is adequately described — descriptive statistics including means, standard deviations, and frequencies are specified, and these are appropriately linked to the joint display. However, the protocol does not sufficiently describe how quantitative results will inform the qualitative phase beyond the general statement in Table 1 that findings will be used "to inform adjustment of initial interview questions and probes." In an explanatory sequential design, this connection is not a minor procedural detail — it is the defining feature of the design itself. The authors should specify which quantitative findings (e.g., domains with notably low or high frequencies, patterns associated with extreme well-being scores) will trigger adjustments to the interview guide, and by what criteria those adjustments will be made. Without this, the sequential and explanatory logic of the design remains asserted but not demonstrated. | Thanks for pointing that out. Please see a revision on page 11 |
| 2. Mixed-methods integration remains descriptive, not analytical The statistics-by-themes joint display describes how data will be organized, not how it will be interpreted. The manuscript lacks a procedural account of how the research team will reason through convergences and divergences to generate meta-inferences. The authors should describe this step-by-step interpretive process explicitly, including the criteria used to determine whether qualitative findings confirm, expand, or contradict quantitative results. The authors may find it useful to reference Fetters et al. (2013) or Creswell & Plano Clark (2018) as frameworks for structuring this description. | We appreciate this excellent methodological critique. We agree that our previous description outlined the organizational structure of the joint display without detailing the rigorous, step-by-step analytical reasoning required to generate meta-inferences. Please see page 11. |
| For the integration process to be replicable, the protocol must include a concrete description of the reasoning chain. For instance: if quantitative results show that women with low well-being scores report high frequency of economic instability (Domain 1), and qualitative themes from that same group reveal narratives of treatment non-adherence due to financial barriers, how would the team determine whether this constitutes convergence, and what integrated conclusion — one that neither strand could produce alone — would be drawn from that pattern? This level of methodological detail is necessary for the mixed-methods integration to meet publication standards as a protocol. | Thanks for pointing that out. Please see a revision on page 11 |
| 3. Cultural validity of the SDoH instrument is insufficiently justified The cited reference (Chunara et al., 2024, [4]) is a methodological commentary on data science and does not validate any questionnaire or reference Thai populations. A CVI from expert review establishes content relevance, not cultural equivalence. The authors should either cite a validation study in a Thai or comparable Southeast Asian population, or provide a methodological justification for applying Healthy People 2030 domains to the Thai sociocultural context supported by relevant literature. If neither can be established, this should be explicitly acknowledged as a limitation in the Discussion section, addressing its potential implications for the interpretation of SDoH findings. | Thanks! We have added a limitation on page 12. |

| **Reviewer #6 comments** | **Authors’ Response** |
| --- | --- |
| The revised manuscript covered all my previous comments. I do have new comments on it. The protocol. now reads good to me | Thank you very much! |

We appreciate your thoughtful comments and suggestions. We believe that through the revision process; the manuscript has been improved. Thank you in advance for your consideration.

We look forward to hearing from you.

Sincerely,

Authors
