# Supplementary material for: Social determinants of health and postnatal wellbeing among women with type 2 diabetes in Thailand: An explanatory sequential mixed-methods study protocol
Source: PLoS One. 2026 Jul 29;21(7):e0354735. doi: 10.1371/journal.pone.0354735 (PMC13419172; doi:10.1371/journal.pone.0354735)
Supplement: Peer review history

# Peer review: Decision Letter 0

**DOI:** 10.1371/journal.pone.0354735.r001

**Authors:** Sandra India Aldana

15 Apr 2026

PONE-D-25-52097

Social Determinants of Health and Postnatal Wellbeing among Women with Type 2 Diabetes in Thailand: An Explanatory Sequential Mixed-Methods Study protocol

PLOS One

Dear Dr. Srichalerm,

Thank you for submitting your manuscript to PLOS ONE. After careful consideration, we feel that it has merit but does not fully meet PLOS ONE’s publication criteria as it currently stands. Therefore, we invite you to submit a revised version of the manuscript that addresses the points raised during the review process.

Please submit your revised manuscript by May 30 2026 11:59PM. If you will need more time than this to complete your revisions, please reply to this message or contact the journal office at plosone@plos.org. When you're ready to submit your revision, log on to https://www.editorialmanager.com/pone/ and select the 'Submissions Needing Revision' folder to locate your manuscript file.

Please include the following items when submitting your revised manuscript:

- A letter that responds to each point raised by the academic editor and reviewer(s). You should upload this letter as a separate file labeled 'Response to Reviewers'.
- A marked-up copy of your manuscript that highlights changes made to the original version. You should upload this as a separate file labeled 'Revised Manuscript with Track Changes'.
- An unmarked version of your revised paper without tracked changes. You should upload this as a separate file labeled 'Manuscript'.

If you would like to make changes to your financial disclosure, please include your updated statement in your cover letter. Guidelines for resubmitting your figure files are available below the reviewer comments at the end of this letter.

If applicable, we recommend that you deposit your laboratory protocols in protocols.io to enhance the reproducibility of your results. Protocols.io assigns your protocol its own identifier (DOI) so that it can be cited independently in the future. For instructions see: https://journals.plos.org/plosone/s/submission-guidelines#loc-laboratory-protocols. Additionally, PLOS ONE offers an option for publishing peer-reviewed Lab Protocol articles, which describe protocols hosted on protocols.io. Read more information on sharing protocols at https://plos.org/protocols?utm_medium=editorial-email&utm_source=authorletters&utm_campaign=protocols.

As the corresponding author, your ORCID iD is verified in the submission system and will appear in the published article. PLOS supports the use of ORCID, and we encourage all coauthors to register for an ORCID iD and use it as well. Please encourage your coauthors to verify their ORCID iD within the submission system before final acceptance, as unverified ORCID iDs will not appear in the published article. *Only*  the individual author can complete the verification step; PLOS staff *cannot*  verify ORCID iDs on behalf of authors.

We look forward to receiving your revised manuscript.

Kind regards,

Sandra India Aldana, MPH, MPhil, PhD

Guest Editor

PLOS One

Journal Requirements:

When submitting your revision, we need you to address these additional requirements.

1. Please ensure that your manuscript meets PLOS ONE's style requirements, including those for file naming. The PLOS ONE style templates can be found at

https://journals.plos.org/plosone/s/file?id=wjVg/PLOSOne_formatting_sample_main_body.pdf and

https://journals.plos.org/plosone/s/file?id=ba62/PLOSOne_formatting_sample_title_authors_affiliations.pdf

2. Thank you for stating the following in your Competing Interests section:

"None"

Please complete your Competing Interests on the online submission form to state any Competing Interests. If you have no competing interests, please state "The authors have declared that no competing interests exist.", as detailed online in our guide for authors at http://journals.plos.org/plosone/s/submit-now

This information should be included in your cover letter; we will change the online submission form on your behalf.

3. Your ethics statement should only appear in the Methods section of your manuscript. If your ethics statement is written in any section besides the Methods, please move it to the Methods section and delete it from any other section. Please ensure that your ethics statement is included in your manuscript, as the ethics statement entered into the online submission form will not be published alongside your manuscript.

4. We note that you have indicated that there are restrictions to data sharing for this study. For studies involving human research participant data or other sensitive data, we encourage authors to share de-identified or anonymized data. However, when data cannot be publicly shared for ethical reasons, we allow authors to make their data sets available upon request. For information on unacceptable data access restrictions, please see http://journals.plos.org/plosone/s/data-availability#loc-unacceptable-data-access-restrictions.

Before we proceed with your manuscript, please address the following prompts:

a) If there are ethical or legal restrictions on sharing a de-identified data set, please explain them in detail (e.g., data contain potentially identifying or sensitive patient information, data are owned by a third-party organization, etc.) and who has imposed them (e.g., a Research Ethics Committee or Institutional Review Board, etc.). Please also provide contact information for a data access committee, ethics committee, or other institutional body to which data requests may be sent.

b) If there are no restrictions, please upload the minimal anonymized data set necessary to replicate your study findings to a stable, public repository and provide us with the relevant URLs, DOIs, or accession numbers. Please see http://www.bmj.com/content/340/bmj.c181.long for guidelines on how to de-identify and prepare clinical data for publication. For a list of recommended repositories, please see https://journals.plos.org/plosone/s/recommended-repositories. You also have the option of uploading the data as Supporting Information files, but we would recommend depositing data directly to a data repository if possible.

Please update your Data Availability statement in the submission form accordingly.

5. If the reviewer comments include a recommendation to cite specific previously published works, please review and evaluate these publications to determine whether they are relevant and should be cited. There is no requirement to cite these works unless the editor has indicated otherwise.

Additional Editor Comments:

See comments below. See also attached the materials provided by reviewer(s) whenever they are provided, if helpful.

[Note: HTML markup is below. Please do not edit.]

Reviewers' comments:

Reviewer's Responses to Questions

**Comments to the Author**

1. Does the manuscript provide a valid rationale for the proposed study, with clearly identified and justified research questions?

The research question outlined is expected to address a valid academic problem or topic and contribute to the base of knowledge in the field.

Reviewer #1: Yes

Reviewer #2: Partly

Reviewer #3: Yes

Reviewer #4: Yes

Reviewer #5: Yes

Reviewer #6: Yes

**********

2. Is the protocol technically sound and planned in a manner that will lead to a meaningful outcome and allow testing the stated hypotheses?

The manuscript should describe the methods in sufficient detail to prevent undisclosed flexibility in the experimental procedure or analysis pipeline, including sufficient outcome-neutral conditions (e.g. necessary controls, absence of floor or ceiling effects) to test the proposed hypotheses and a statistical power analysis where applicable. As there may be aspects of the methodology and analysis which can only be refined once the work is undertaken, authors should outline potential assumptions and explicitly describe what aspects of the proposed analyses, if any, are exploratory.

Reviewer #1: Partly

Reviewer #2: Partly

Reviewer #3: Yes

Reviewer #4: Yes

Reviewer #5: No

Reviewer #6: Yes

**********

3. Is the methodology feasible and described in sufficient detail to allow the work to be replicable?

Descriptions of methods and materials in the protocol should be reported in sufficient detail for another researcher to reproduce all experiments and analyses. The protocol should describe the appropriate controls, sample size calculations, and replication needed to ensure that the data are robust and reproducible.

Reviewer #1: Yes

Reviewer #2: No

Reviewer #3: Yes

Reviewer #4: Yes

Reviewer #5: No

Reviewer #6: Yes

**********

4. Have the authors described where all data underlying the findings will be made available when the study is complete?

The PLOS Data policy requires authors to make all data underlying the findings described in their manuscript fully available without restriction, with rare exception, at the time of publication. The data should be provided as part of the manuscript or its supporting information, or deposited to a public repository. For example, in addition to summary statistics, the data points behind means, medians and variance measures should be available. If there are restrictions on publicly sharing data—e.g. participant privacy or use of data from a third party—those must be specified.

Reviewer #1: Yes

Reviewer #2: Yes

Reviewer #3: Yes

Reviewer #4: Yes

Reviewer #5: No

Reviewer #6: No

**********

5. Is the manuscript presented in an intelligible fashion and written in standard English?

PLOS ONE does not copyedit accepted manuscripts, so the language in submitted articles must be clear, correct, and unambiguous. Any typographical or grammatical errors should be corrected at revision, so please note any specific errors here.

Reviewer #1: Yes

Reviewer #2: Yes

Reviewer #3: Yes

Reviewer #4: Yes

Reviewer #5: Yes

Reviewer #6: Yes

**********

6. Review Comments to the Author

Please use the space provided to explain your answers to the questions above and, if applicable, provide comments about issues authors must address before this protocol can be accepted for publication. You may also include additional comments for the author, including concerns about research or publication ethics.

You may also provide optional suggestions and comments to authors that they might find helpful in planning their study.

(Please upload your review as an attachment if it exceeds 20,000 characters)

Reviewer #1: General evaluation: the author presents an interesting Sequential Explanatory Mixed Methods (QUAN; qual) study addressing women’s health in the Thai context. The integration of REDCap for data management and the hybrid thematic analysis approach are strengths. However, there are methodological discrepancies and terminological inconsistencies, particularly regarding instrument validation and sampling logic, that must be addressed to meet the standards of this journal. 1. Content Validity Index (CVI) and Expert Panel Size. The author states that the questionnaires were validated by a panel of three experts. While three is the absolute minimum, it poses a statistical risk. According to Lynn (1986) and Polit & Beck, with a panel of three, the required I-CVI for an item to be considered valid must be 1.00 (100% agreement). Furthermore, if any items were modified based on expert feedback, please describe the iterative process. For a study of this scope, a larger panel (5–10 experts) is preferred to ensure robustness. The protocol lists the "Interview Guide" as being translated and validated via CVI during the initial Quantitative Phase. However, the subsequent section correctly notes that the qualitative probes will be informed by the quantitative results.

Reviewer #2: SUMMARY OF REVIEW COMMENTS - MANUSCRIPT NUMBER: PONE-D-25-52097

Here I present a summary of recommendations; the full document with detailed comments and recommendations is attached as a PDF file.

This manuscript addresses a highly relevant and meaningful topic, focusing on social determinants of health and postnatal well-being among women with type 2 diabetes, an area where further evidence is clearly needed to inform clinical practice and health policy. The study has the potential to contribute valuable insights, particularly given its mixed-methods approach and its focus on a specific population and context. My comments are provided in the spirit of strengthening the manuscript, with particular emphasis on improving clarity of reporting, methodological transparency, and reproducibility. These aspects are especially important in protocol papers, as they allow readers to understand the planned study in detail and to assess any deviations when the final results are published. For clarity, my comments and recommendations are organized into two sections: (1) Introduction and (2) Methods. Each section concludes with a summary of key recommendations to facilitate revision.

Summary of recommendation for the introduction

Overall, the introduction would benefit from (1) reorganizing the sequence of ideas to clearly build the research gap and study rationale; (2) removing repeated statements and redundancies, particularly in the description of postpartum challenges and care needs; (3) presenting the aim and specific objectives in a consistent and coherent structure and in a single location; (4) clearly aligning the background with the study scope, the use of the Postnatal Well-being in Transition instrument, and the mixed-methods design, with explicit justification of these choices; (5) providing a clear and explicit rationale for the selection of T2DM as the study population, including its relevance to this age group and context; and (6) improving the precision of key statements and ensuring appropriate alignment between claims and supporting references, prioritizing direct empirical or primary sources over secondary or conceptual citations where aplicable; and 7) Consider removing one of the two elements to avoid redundancy and relocate the remaining figure/table to the methods section, where it more appropriately supports the study design description.

Summary of recommendation for the method section:

The Methods section would benefit from substantial revision to:

(1) restructure the section to improve logical flow and eliminate repetition, ensuring that each component of the study is presented once and in a clear sequence; if appropriate, the authors may consider following established guidelines for mixed-methods reporting, such as the Good Reporting of A Mixed Methods Study (GRAMMS) or the Mixed Methods Appraisal Tool (MMAT).

(2) clarify the status of the study (planned, ongoing, or partially completed), including consistency between timelines and reported completed steps, and ensure consistent use of verb tense throughout;

(3) streamline the description of the study design to avoid redundancy with the Introduction and clearly distinguish between conceptual rationale and methodological implementation;

(4) improve the description and justification of the research setting, including specifying locations and relevant characteristics, and reconsider claims such as “maximum variation” in light of the actual sampling scope;

(5) simplify and accurately describe the sampling strategy, using appropriate and consistent methodological terminology and clearly explaining how participants are selected in practice;

(6) ensure the correct use and explanation of methodological concepts (e.g., sampling strategies, back-translation), avoiding conceptual inaccuracies and inconsistencies;

(7) provide stronger and more appropriate justification for both quantitative and qualitative sample sizes, avoiding the treatment of saturation as a fixed number and ensuring alignment with qualitative research principles;

(8) describe the characteristics, structure, adaptation, and validation of all instruments—particularly the SDoH measure and the translated questionnaire—in sufficient detail to allow assessment of their appropriateness, including clarification of whether instruments were adapted or newly developed and whether translation/validation has been completed;

(9) ensure that the reported procedures are clearly aligned with established methodological frameworks (e.g., Beaton guidelines), with transparent description of each step;

(10) ensure consistency across the text, figures, and tables (e.g., in the description of study phases);

(11) reduce redundancy in methodological descriptions, particularly regarding data collection, analysis, and integration, and avoid repetition of general methodological concepts;

(12) enhance transparency and reproducibility by considering inclusion of relevant materials (e.g., translated instrument, validation results) as supplementary files; and

(13) revise all in-text citations to comply with PLOS guidelines, ensuring accurate correspondence with the reference list.

Reviewer #3: The authors provided a valid rationale for the propose study.Also the protocol is technically planned, with a visible methodology.

Reviewer #4: Review of Manuscript: Social Determinants of Health and Postnatal Wellbeing among Women with Type 2 Diabetes in Thailand: An Explanatory Sequential Mixed-Methods Study protocol

This manuscript presents a protocol for an explanatory sequential mixed-methods study examining the influence of social determinants of health (SDOH) on postnatal wellbeing among women with Type 2 diabetes in Thailand. The study addresses an important and underexplored area at the intersection of maternal health, chronic disease, and social determinants. The mixed-methods design is appropriate to the research question, allowing quantitative assessment followed by qualitative exploration. Overall, the protocol is of potential value.

I am giving my observations and comments for the author to consider:

• Introduction: The abbreviation used is to be consistent. Suggest the author to stick to either SDOH or SDoH

• Line 70 – Does the author mean extensive research exist on SDoH impact on diabetes in pregnancy?

• Line 77-79 - The statement is bit confusing, the difficulties mentioned included delivery but says after childbirth, may suggest to rewrite the statement for clarity

Quantitative Instrument and Validation

• Objectives of the study were clearly stated.

• The description of the translation and validation process (Lines 157–159) requires clarification. It should be explicitly stated whether validation has been completed or is planned.

• Additional details are needed on the Content Validity Index (CVI), including:

o Methods used to calculate item-level (I-CVI) and scale-level (S-CVI)

o Criteria for item retention or removal

o The process of instrument refinement (e.g., number of items modified or excluded) should be described.

• The manuscript should include a citation for the “Postnatal Wellbeing in Transition” questionnaire

Qualitative Sampling and Description

• While the linkage between quantitative and qualitative phases is described, the authors should more clearly specify the type(s) of purposive sampling employed (e.g., maximum variation, criterion-based) and the rationale for their use. The paragraph pertaining to it be more refined

Integration of Mixed Methods

• The triangulation is mentioned and described

• The integration between quantitative and qualitative findings are explained

Minor Comments

Ensure consistent terminology throughout (e.g., “postnatal” vs. “postpartum”). Overall the manuscript would benefit from minor language editing to improve readability.

Reviewer #5: This research protocol proposes an explanatory sequential mixed-methods study to explore the social determinants of health (SDoH) and postnatal well-being among women with type 2 diabetes mellitus (T2DM) in Thailand. The study aims to translate the Postnatal Well-being in Transition scale into Thai , quantitatively assess SDoH and well-being , qualitatively explore experiences based on well-being scores , and analyze the impact of postnatal care using a mixed-methods approach. The protocol outlines a two-phase study starting with a quantitative cross-sectional design involving 50 postpartum women , followed by semi-structured interviews with 12 selected participants to deepen the understanding of their transition to motherhood. While the study addresses a critical gap in maternity care and population health, there are substantial methodological and operational details currently missing.

I would recommend a Major Revision focused on rigorously defining the statistical and operational aspects of the study design, particularly regarding the evaluation of "postnatal care" and the exact linkages between the quantitative and qualitative sampling algorithms. Implementing the suggested revisions would increase the protocol’s reproducibility, transparency, and scientific rigor.

Major Methodological & Statistical Issues

1. Lack of Operationalization for "Postnatal Care" Impact (Aim 4) The protocol states an objective to "explore and analyze the impact of postnatal care on women with type 2 diabetes". [Lines 110-111]

Critique: The Methods section does not define or measure 'postnatal care' as an exposure or variable, does not describe any comparator groups or a pre/post design, and provides no statistical or qualitative analytic plan intended to estimate or assess impact. Without operationalization of postnatal care and an analytic approach, there is no executable path from the data to an impact estimate.

Recommendation: The authors must explicitly define how 'postnatal care' will be operationalized and measured. Please specify any comparator groups or exposure levels, and detail the exact statistical or mixed-method analytic approach that will be used to assess its impact.

2. Incomplete Quantitative Endpoints and Analysis Plan The quantitative phase mentions recruiting 50 participants and performing descriptive statistics [232-235].

Critique: The protocol lacks a prespecified quantitative analysis plan, including primary endpoint definitions and statistical tests.

Recommendation: Specify one or more primary endpoint(s) and the corresponding quantitative analysis and statistical tests. Provide a quantitative sample-size justification (power or precision). Prespecify the quantitative analysis plan for missing data handling, covariates/adjustments, and any intended subgroup or sensitivity analyses.

3. Ambiguous Qualitative Sampling Algorithm The protocol states that 12 women will be selected for interviews—six with the lowest scores and six with the highest scores from each of six subscales [Lines 183-189].

Critique: This description is mathematically ambiguous and contradictory. Selecting six from each of six subscales equals 36 high + 36 low = 72 individuals, which conflicts with the stated N=12 total. Additionally, a clear method for integrating these scores with the qualitative themes is missing.

Recommendation: Clarify the exact selection rules and the algorithm used to select exactly 12 qualitative interviewees from the quantitative scores, providing accurate sample counts. Specify a reproducible analytic method for linking qualitative themes to participants' mean scores.

4. Superficial Mixed-Methods Integration (Triangulation) The protocol states that a side-by-side joint display will be used to compare and contrast the results for triangulation [Lines 279-283].

Critique: Merely presenting qualitative themes next to quantitative statistics constitutes data reporting, not full methodological integration. The protocol lacks a clear explanation of how these two strands will interact analytically.

Recommendation: Expand on the analytical process for data integration. Beyond placing data side-by-side, please detail the specific procedures for how the qualitative findings will be explicitly used to expand upon, explain, or contextualize the convergence and divergence found in the quantitative data to generate robust meta-inferences.

5. Theoretical Framework Congruence and Missing SDoH Tool Justification The protocol bases its SDoH domains on Healthy People 2030. [Introduction section and Lines 105 - 121]

Critique: Healthy People 2030 is a US-centric framework. The manuscript currently lacks the necessary bibliography or methodological description demonstrating that the specific SDoH instrument chosen has been validated or culturally adapted for use in Thailand. While Objective 1 explicitly details the translation and adaptation of the Postnatal Well-being in Transition scale, similar reporting is absent for the SDoH tool.

Recommendation: Please provide appropriate citations demonstrating the validity and reliability of the chosen SDoH questionnaire within the Thai population. If the tool has not been previously validated in this context, clarify whether a cultural adaptation process will be conducted, or justify methodologically why the US-centric domains are directly applicable to the Thai socio-cultural and healthcare setting without modification.

6. Qualitative Rigor: Positionality and Reflexivity The protocol details the interview process but omits researcher details [Lines 182 - 212].

Critique: Interviewer bias and power dynamics play a critical role in data quality. If healthcare personnel from the participants' own hospital settings conduct the interviews, patients might not provide honest assessments of their well-being or care due to social desirability bias or fear of negative repercussions on their clinical treatment.

Recommendation: Please include a reflexivity statement addressing the researchers' positionality (specifying the professional background and clinical role of the interviewers). Detail the specific strategies employed to mitigate power dynamics between the interviewer and the participants.

Specific & Minor Comments

* Recruitment & Bias: Clarify the recruitment and enrollment workflow, including online consent procedures and site-specific recruitment targets across the urban and suburban hospitals. Describe concrete bias-mitigation measures (e.g., consecutive or stratified recruitment, mode equivalence checks, monitoring nonresponse) and explain how the representativeness of the sample will be assessed.

* Translation Procedures: Report the qualifications of the forward and back translators and summarize any pilot testing procedures and results used to finalize the Thai version.

* SDoH Instrumentation: Provide the full SDoH instrument items or exact references, and specifically state how each SDoH domain and the postnatal well-being scale will be scored and operationalized for the planned descriptive analyses.

Reviewer #6: 1. Line 59: A typo in “social determinants of health (SDOH)”, should be “SDoH”.

2. Part 2 Study data collection: Will information of delivery mode (C-section, virginal, etc.), blood glucose during pregnancy and postpartum period, medication data, and baby information (gestational age, birth weight, etc.) be collected or be available through medical records?

3. Lines 200-205: The authors mentioned that based on the quantitative phase results, stratified sampling approach will be used to select samples for qualitative interview. It is vague and unclear what subgroups will be here. In the first paragraph of this section, they mentioned that 6 participants with the lowest scores and highest scores will be interviewed, how this will be incorporated in the potentially stratified sampling approach.

4. Lines 219-221: The authors mentioned that they will assess SDoH covering 5 domains and stated that they will apply for questionnaires from previous research with ref. [4]. Ref [4] is a review article on racial and ethnic disparities in health care and health outcomes for pregnancy women with diabetes, it cannot cover all the 5 domains. I would appreciate it if the authors can list the validated questionnaires of SDoH that they plan to use in their forth coming survey.

5. Lines 275-278: The authors mentioned that “to conduct triangulation, qualitative and quantitative analyses will be conducted separately”, but it seems that the team adapted a sequential approach that the qualitative samples will be selected based on quantitative scores, then how to make sure that the triangulation process is not bias because this nested sampling strategy?

6. In the data management section, please provide data sharing statement on how will the collected data be published or will data be available upon request.

**********

7. PLOS authors have the option to publish the peer review history of their article (what does this mean?). If published, this will include your full peer review and any attached files.

If you choose “no”, your identity will remain anonymous but your review may still be made public.

**Do you want your identity to be public for this peer review?**  For information about this choice, including consent withdrawal, please see our Privacy Policy.

Reviewer #1: No

Reviewer #2: **Yes:**  Steven Cuadra

Reviewer #3: **Yes:**  ANTHONY DIATUO

Reviewer #4: No

Reviewer #5: **Yes:**  Juan Rodrigo Gomez-Bernal

Reviewer #6: No

**********

[NOTE: If reviewer comments were submitted as an attachment file, they will be attached to this email and accessible via the submission site. Please log into your account, locate the manuscript record, and check for the action link "View Attachments". If this link does not appear, there are no attachment files.]

To ensure your figures meet our technical requirements, please review our figure guidelines: https://journals.plos.org/plosone/s/figures

You may also use PLOS’s free figure tool, NAAS, to help you prepare publication quality figures: https://journals.plos.org/plosone/s/figures#loc-tools-for-figure-preparation.

NAAS will assess whether your figures meet our technical requirements by comparing each figure against our figure specifications.

Attachment Submitted filename: Review Manuscript PONE-D-25-52097.pdf

# Author comment: Author response to Decision Letter 1

**DOI:** 10.1371/journal.pone.0354735.r002

27 Apr 2026

Dear Reviewers,

Thank you for dedicating your time and effort to providing valuable feedback on our work.

We have addressed the editor's and reviewers’ comments and concerns in a point-by-point manner. Please find our responses to each of them outlined below. We believe the manuscript has been considerably strengthened with these revisions.

Best regards,

Attachment Submitted filename: Response to reviewers.docx

# Peer review: Decision Letter 1

**DOI:** 10.1371/journal.pone.0354735.r003

**Authors:** Sandra India Aldana

26 May 2026

PONE-D-25-52097R1

Social Determinants of Health and Postnatal Wellbeing among Women with Type 2 Diabetes in Thailand: An Explanatory Sequential Mixed-Methods Study protocol

PLOS One

Dear Dr. Srichalerm,

Thank you for submitting your manuscript to PLOS ONE. After careful consideration, we feel that it has merit but does not fully meet PLOS ONE’s publication criteria as it currently stands. Therefore, we invite you to submit a revised version of the manuscript that addresses the points raised during the review process.

Please submit your revised manuscript by Jul 09 2026 11:59PM. If you will need more time than this to complete your revisions, please reply to this message or contact the journal office at plosone@plos.org. When you're ready to submit your revision, log on to https://www.editorialmanager.com/pone/ and select the 'Submissions Needing Revision' folder to locate your manuscript file.

Please include the following items when submitting your revised manuscript:

- A letter that responds to each point raised by the academic editor and reviewer(s). You should upload this letter as a separate file labeled 'Response to Reviewers'.
- A marked-up copy of your manuscript that highlights changes made to the original version. You should upload this as a separate file labeled 'Revised Manuscript with Track Changes'.
- An unmarked version of your revised paper without tracked changes. You should upload this as a separate file labeled 'Manuscript'.

If you would like to make changes to your financial disclosure, please include your updated statement in your cover letter. Guidelines for resubmitting your figure files are available below the reviewer comments at the end of this letter.

If applicable, we recommend that you deposit your laboratory protocols in protocols.io to enhance the reproducibility of your results. Protocols.io assigns your protocol its own identifier (DOI) so that it can be cited independently in the future. For instructions see: https://journals.plos.org/plosone/s/submission-guidelines#loc-laboratory-protocols. Additionally, PLOS ONE offers an option for publishing peer-reviewed Lab Protocol articles, which describe protocols hosted on protocols.io. Read more information on sharing protocols at https://plos.org/protocols?utm_medium=editorial-email&utm_source=authorletters&utm_campaign=protocols.

As the corresponding author, your ORCID iD is verified in the submission system and will appear in the published article. PLOS supports the use of ORCID, and we encourage all coauthors to register for an ORCID iD and use it as well. Please encourage your coauthors to verify their ORCID iD within the submission system before final acceptance, as unverified ORCID iDs will not appear in the published article. *Only*  the individual author can complete the verification step; PLOS staff *cannot*  verify ORCID iDs on behalf of authors.

We look forward to receiving your revised manuscript.

Kind regards,

Sandra India Aldana, MPH, MPhil, PhD

Guest Editor

PLOS One

Journal Requirements:

1. If the reviewer comments include a recommendation to cite specific previously published works, please review and evaluate these publications to determine whether they are relevant and should be cited. There is no requirement to cite these works unless the editor has indicated otherwise.

Additional Editor Comments:

Reviewer 1

The authors have addressed all the comments and concerns raised in the previous review round. The paper is now clear, scientifically sound. I have no further comments.

Reviewer 4

The authors have adequately addressed most of the comments raised and the manuscript has improved substantially in terms of detailing and clarity. However there are few minor issues to consider:

Exclusion criteria: it is not clear whether the clinical status or outcome of the baby (e.g., neonatal complications, NICU admission, stillbirth, neonatal death, congenital anomalies, etc.) will influence participant selection. This may be explicitly stated.

The study objectives and the research phases are still not clearly aligned as previously commented by the reviewers. The translation of the instrument which was pointed out earlier by a reviewer as a methodological step rather than a study objective and may be included as part of the quantitative phase is still listed as the first aim.

Appreciate that the domains included in the questionnaire are described. However information regarding the number of questions/items under each domain is missing.

The manuscript mentions that data collection will continue until December 2026; however, the study is simultaneously described as completed in the following sentence. Please clarify

In Table 1: The Interview guide may be moved to as part of Qualitative phase

Reviewer 5

I appreciate the authors' efforts in revising the manuscript. Several improvements are noted, including the clarification of the qualitative sampling algorithm and the expanded recruitment description. However, three critical concerns remain unresolved.

1. Objective lacks analytical operationalization

Reformulating the objective from "evaluate impact" to "evaluate postnatal well-being using a mixed-methods approach" does not resolve the original concern. Insufficient description of the quantitative-to-qualitative transition

The quantitative analysis plan is adequately described — descriptive statistics including means, standard deviations, and frequencies are specified, and these are appropriately linked to the joint display. However, the protocol does not sufficiently describe how quantitative results will inform the qualitative phase beyond the general statement in Table 1 that findings will be used "to inform adjustment of initial interview questions and probes." In an explanatory sequential design, this connection is not a minor procedural detail — it is the defining feature of the design itself. The authors should specify which quantitative findings (e.g., domains with notably low or high frequencies, patterns associated with extreme well-being scores) will trigger adjustments to the interview guide, and by what criteria those adjustments will be made. Without this, the sequential and explanatory logic of the design remains asserted but not demonstrated.

2. Mixed-methods integration remains descriptive, not analytical

The statistics-by-themes joint display describes how data will be organized, not how it will be interpreted. The manuscript lacks a procedural account of how the research team will reason through convergences and divergences to generate meta-inferences. The authors should describe this step-by-step interpretive process explicitly, including the criteria used to determine whether qualitative findings confirm, expand, or contradict quantitative results. The authors may find it useful to reference Fetters et al. (2013) or Creswell & Plano Clark (2018) as frameworks for structuring this description.

For the integration process to be replicable, the protocol must include a concrete description of the reasoning chain. For instance: if quantitative results show that women with low well-being scores report high frequency of economic instability (Domain 1), and qualitative themes from that same group reveal narratives of treatment non-adherence due to financial barriers, how would the team determine whether this constitutes convergence, and what integrated conclusion — one that neither strand could produce alone — would be drawn from that pattern? This level of methodological detail is necessary for the mixed-methods integration to meet publication standards as a protocol.

3. Cultural validity of the SDoH instrument is insufficiently justified

The cited reference (Chunara et al., 2024, [4]) is a methodological commentary on data science and does not validate any questionnaire or reference Thai populations. A CVI from expert review establishes content relevance, not cultural equivalence. The authors should either cite a validation study in a Thai or comparable Southeast Asian population, or provide a methodological justification for applying Healthy People 2030 domains to the Thai sociocultural context supported by relevant literature. If neither can be established, this should be explicitly acknowledged as a limitation in the Discussion section, addressing its potential implications for the interpretation of SDoH findings.

Reviewer 6

The revised manuscript covered all my previous comments. I do have new comments on it. The protocol. now reads good to me

[Note: HTML markup is below. Please do not edit.]

Reviewer's Responses to Questions

**Comments to the Author**

1. Does the manuscript provide a valid rationale for the proposed study, with clearly identified and justified research questions?

The research question outlined is expected to address a valid academic problem or topic and contribute to the base of knowledge in the field.

Reviewer #1: Yes

Reviewer #4: Yes

Reviewer #5: Partly

Reviewer #6: Yes

**********

2. Is the protocol technically sound and planned in a manner that will lead to a meaningful outcome and allow testing the stated hypotheses?

The manuscript should describe the methods in sufficient detail to prevent undisclosed flexibility in the experimental procedure or analysis pipeline, including sufficient outcome-neutral conditions (e.g. necessary controls, absence of floor or ceiling effects) to test the proposed hypotheses and a statistical power analysis where applicable. As there may be aspects of the methodology and analysis which can only be refined once the work is undertaken, authors should outline potential assumptions and explicitly describe what aspects of the proposed analyses, if any, are exploratory.

Reviewer #1: Yes

Reviewer #4: Yes

Reviewer #5: Partly

Reviewer #6: Yes

**********

3. Is the methodology feasible and described in sufficient detail to allow the work to be replicable?

Descriptions of methods and materials in the protocol should be reported in sufficient detail for another researcher to reproduce all experiments and analyses. The protocol should describe the appropriate controls, sample size calculations, and replication needed to ensure that the data are robust and reproducible.

Reviewer #1: Yes

Reviewer #4: Yes

Reviewer #5: Yes

Reviewer #6: Yes

**********

4. Have the authors described where all data underlying the findings will be made available when the study is complete?

The PLOS Data policy requires authors to make all data underlying the findings described in their manuscript fully available without restriction, with rare exception, at the time of publication. The data should be provided as part of the manuscript or its supporting information, or deposited to a public repository. For example, in addition to summary statistics, the data points behind means, medians and variance measures should be available. If there are restrictions on publicly sharing data—e.g. participant privacy or use of data from a third party—those must be specified.

Reviewer #1: Yes

Reviewer #4: Yes

Reviewer #5: Yes

Reviewer #6: Yes

**********

5. Is the manuscript presented in an intelligible fashion and written in standard English?

PLOS ONE does not copyedit accepted manuscripts, so the language in submitted articles must be clear, correct, and unambiguous. Any typographical or grammatical errors should be corrected at revision, so please note any specific errors here.

Reviewer #1: Yes

Reviewer #4: Yes

Reviewer #5: Yes

Reviewer #6: Yes

**********

6. Review Comments to the Author

Please use the space provided to explain your answers to the questions above and, if applicable, provide comments about issues authors must address before this protocol can be accepted for publication. You may also include additional comments for the author, including concerns about research or publication ethics.

You may also provide optional suggestions and comments to authors that they might find helpful in planning their study.

(Please upload your review as an attachment if it exceeds 20,000 characters)

Reviewer #1: The authors have addressed all the comments and concerns raised in the previous review round. The paper is now clear, scientifically sound. I have no further comments.

Reviewer #4: The authors have adequately addressed most of the comments raised and the manuscript has improved substantially in terms of detailing and clarity. However there are few minor issues to consider:

Exclusion criteria: it is not clear whether the clinical status or outcome of the baby (e.g., neonatal complications, NICU admission, stillbirth, neonatal death, congenital anomalies, etc.) will influence participant selection. This may be explicitly stated.

The study objectives and the research phases are still not clearly aligned as previously commented by the reviewers. The translation of the instrument which was pointed out earlier by a reviewer as a methodological step rather than a study objective and may be included as part of the quantitative phase is still listed as the first aim.

Appreciate that the domains included in the questionnaire are described. However information regarding the number of questions/items under each domain is missing.

The manuscript mentions that data collection will continue until December 2026; however, the study is simultaneously described as completed in the following sentence. Please clarify

In Table 1: The Interview guide may be moved to as part of Qualitative phase

Recommendation: Accept with minor revision

Reviewer #5: I appreciate the authors' efforts in revising the manuscript. Several improvements are noted, including the clarification of the qualitative sampling algorithm and the expanded recruitment description. However, three critical concerns remain unresolved.

1. Objective lacks analytical operationalization

Reformulating the objective from "evaluate impact" to "evaluate postnatal well-being using a mixed-methods approach" does not resolve the original concern. Insufficient description of the quantitative-to-qualitative transition

The quantitative analysis plan is adequately described — descriptive statistics including means, standard deviations, and frequencies are specified, and these are appropriately linked to the joint display. However, the protocol does not sufficiently describe how quantitative results will inform the qualitative phase beyond the general statement in Table 1 that findings will be used "to inform adjustment of initial interview questions and probes." In an explanatory sequential design, this connection is not a minor procedural detail — it is the defining feature of the design itself. The authors should specify which quantitative findings (e.g., domains with notably low or high frequencies, patterns associated with extreme well-being scores) will trigger adjustments to the interview guide, and by what criteria those adjustments will be made. Without this, the sequential and explanatory logic of the design remains asserted but not demonstrated.

2. Mixed-methods integration remains descriptive, not analytical

The statistics-by-themes joint display describes how data will be organized, not how it will be interpreted. The manuscript lacks a procedural account of how the research team will reason through convergences and divergences to generate meta-inferences. The authors should describe this step-by-step interpretive process explicitly, including the criteria used to determine whether qualitative findings confirm, expand, or contradict quantitative results. The authors may find it useful to reference Fetters et al. (2013) or Creswell & Plano Clark (2018) as frameworks for structuring this description.

For the integration process to be replicable, the protocol must include a concrete description of the reasoning chain. For instance: if quantitative results show that women with low well-being scores report high frequency of economic instability (Domain 1), and qualitative themes from that same group reveal narratives of treatment non-adherence due to financial barriers, how would the team determine whether this constitutes convergence, and what integrated conclusion — one that neither strand could produce alone — would be drawn from that pattern? This level of methodological detail is necessary for the mixed-methods integration to meet publication standards as a protocol.

3. Cultural validity of the SDoH instrument is insufficiently justified

The cited reference (Chunara et al., 2024, [4]) is a methodological commentary on data science and does not validate any questionnaire or reference Thai populations. A CVI from expert review establishes content relevance, not cultural equivalence. The authors should either cite a validation study in a Thai or comparable Southeast Asian population, or provide a methodological justification for applying Healthy People 2030 domains to the Thai sociocultural context supported by relevant literature. If neither can be established, this should be explicitly acknowledged as a limitation in the Discussion section, addressing its potential implications for the interpretation of SDoH findings.

Reviewer #6: The revised manuscript covered all my previous comments. I do have new comments on it. The protocol. now reads good to me

**********

7. PLOS authors have the option to publish the peer review history of their article (what does this mean?). If published, this will include your full peer review and any attached files.

If you choose “no”, your identity will remain anonymous but your review may still be made public.

**Do you want your identity to be public for this peer review?**  For information about this choice, including consent withdrawal, please see our Privacy Policy.

Reviewer #1: No

Reviewer #4: No

Reviewer #5: **Yes:**  Juan Rodrigo Gomez-Bernal

Reviewer #6: No

**********

[NOTE: If reviewer comments were submitted as an attachment file, they will be attached to this email and accessible via the submission site. Please log into your account, locate the manuscript record, and check for the action link "View Attachments". If this link does not appear, there are no attachment files.]

To ensure your figures meet our technical requirements, please review our figure guidelines: https://journals.plos.org/plosone/s/figures

You may also use PLOS’s free figure tool, NAAS, to help you prepare publication quality figures: https://journals.plos.org/plosone/s/figures#loc-tools-for-figure-preparation.

NAAS will assess whether your figures meet our technical requirements by comparing each figure against our figure specifications.

# Author comment: Author response to Decision Letter 2

**DOI:** 10.1371/journal.pone.0354735.r004

7 Jun 2026

Dear Editor and Reviewers,

We appreciate the opportunity to submit our revised manuscript. Thank you for dedicating your time and effort to providing valuable feedback on our work.

We have addressed the editor's and reviewers’ comments and concerns in a point-by-point manner. We believe the manuscript has been considerably strengthened with these revisions.

Kind regards,

Authors

Attachment Submitted filename: Response to reviewers R2.docx

# Peer review: Decision Letter 2

**DOI:** 10.1371/journal.pone.0354735.r005

**Authors:** Sandra India Aldana

12 Jul 2026

Social Determinants of Health and Postnatal Wellbeing among Women with Type 2 Diabetes in Thailand: An Explanatory Sequential Mixed-Methods Study protocol

PONE-D-25-52097R2

Dear Dr. Srichalerm,

We’re pleased to inform you that your manuscript has been judged scientifically suitable for publication and will be formally accepted for publication once it meets all outstanding technical requirements.

Within one week, you’ll receive an e-mail detailing the required amendments. When these have been addressed, you’ll receive a formal acceptance letter and your manuscript will be scheduled for publication.

An invoice will be generated when your article is formally accepted. Please note, if your institution has a publishing partnership with PLOS and your article meets the relevant criteria, all or part of your publication costs will be covered. Please make sure your user information is up-to-date by logging into Editorial Manager at Editorial Manager® and clicking the ‘Update My Information' link at the top of the page. For questions related to billing, please contact billing support.

If your institution or institutions have a press office, please notify them about your upcoming paper to help maximize its impact. If they’ll be preparing press materials, please inform our press team as soon as possible -- no later than 48 hours after receiving the formal acceptance. Your manuscript will remain under strict press embargo until 2 pm Eastern Time on the date of publication. For more information, please contact onepress@plos.org.

Kind regards,

Sandra India Aldana, MPH, MPhil, PhD

Guest Editor

PLOS One

**********

# Editor report: Acceptance letter

**DOI:** 10.1371/journal.pone.0354735.r006

**Authors:** Sandra India Aldana

PONE-D-25-52097R2

PLOS One

Dear Dr. Srichalerm,

I'm pleased to inform you that your manuscript has been deemed suitable for publication in PLOS One. Congratulations! Your manuscript is now being handed over to our production team.

At this stage, our production department will prepare your paper for publication. This includes ensuring the following:

* All references, tables, and figures are properly cited

* All relevant supporting information is included in the manuscript submission,

* There are no issues that prevent the paper from being properly typeset

You will receive further instructions from the production team, including instructions on how to review your proof when it is ready. Please keep in mind that we are working through a large volume of accepted articles, so please give us a few days to review your paper and let you know the next and final steps.

Lastly, if your institution or institutions have a press office, please let them know about your upcoming paper now to help maximize its impact. If they'll be preparing press materials, please inform our press team within the next 48 hours. Your manuscript will remain under strict press embargo until 2 pm Eastern Time on the date of publication. For more information, please contact onepress@plos.org.

You will receive an invoice from PLOS for your publication fee after your manuscript has reached the completed accept phase. If you receive an email requesting payment before acceptance or for any other service, this may be a phishing scheme. Learn how to identify phishing emails and protect your accounts at https://explore.plos.org/phishing.

If we can help with anything else, please email us at customercare@plos.org.

Thank you for submitting your work to PLOS ONE and supporting open access.

Kind regards,

PLOS ONE Editorial Office Staff

on behalf of

Dr. Sandra India Aldana

Guest Editor

PLOS One
